# Dependency of Morphology and Wetting on Alkyl Chain Length in Vacuum-Evaporated [C*_n_* py][NTf_2_] (*n* = 2–9) Pyridinium Ionic Liquid Films

**DOI:** 10.1021/acs.langmuir.6c00729

**Published:** 2026-03-17

**Authors:** Soraia R. M. R. Silva, João M. S. Pereira, Oleksandr Bondarchuk, Mauro C. C. Ribeiro, Luís M. N. B. F. Santos, José C. S. Costa

**Affiliations:** † CIQUP/Institute of Molecular Sciences (IMS), Departamento de Química e Bioquímica, Faculdade de Ciências, Universidade do Porto, Rua do Campo Alegre s/n, Porto 4169-007, Portugal; ‡ 246702 International Iberian Nanotechnology Laboratory, Av. Mestre José Veiga, s/n, Braga 4715-330, Portugal; § SPIN-Lab Centre for Microscopic Research on Matter, University of Silesia in Katowice, 75 Pułku Piechoty Str. 1A, Chorzów 41-500, Poland; ∥ Institute of Chemistry, University of Silesia in Katowice, 9 Szkolna Str., Katowice 40-006, Poland; ⊥ Laboratório de Espectroscopia Molecular, Instituto de Química, 28133 Universidade de São Paulo, São Paulo, SP 5513-970, Brazil

## Abstract

A systematic investigation of thin films of pyridinium-based ionic liquids (ILs), [C*_n_* py]­[NTf_2_] (*n* = 2–9), deposited via physical vapor deposition on ITO and Au/ITO substrates is presented, providing the first comprehensive study of vacuum-deposited films within this homologous series. The influence of evaporation temperature, deposition rate, alkyl chain length, and substrate on thin-film morphology, nucleation and coalescence dynamics, interfacial behavior, and film structure was examined using SEM, optical microscopy, FTIR, and XPS. SEM analyses reveal that higher evaporation temperatures, which increase the deposition rate, lead to larger droplets and enhanced coalescence, resulting in larger microstructures. A comparison of the film morphologies across the IL series shows that longer cation alkyl chains further enhance lateral spreading and wetting, particularly on Au substrates. An odd–even effect on the morphological characteristics of the films is observed across the series, reflecting subtle differences in interfacial interactions. Moreover, a clear distinction in wetting behavior between short- and long-chain pyridinium ILs is evident, consistent with trends previously reported for imidazolium-based ILs. FTIR spectra comparing bulk and thin IL films confirm that the ILs retain their chemical integrity upon film formation. XPS measurements support the morphological observations, highlighting that ILs comprising longer alkyl chains achieve more complete surface coverage. The results of this work provide fundamental insights into the interplay between the cation alkyl chain length of pyridinium-based ILs, substrate interactions, and film formation dynamics, offering guidance for the rational design of IL films for functional surface applications.

## Graphical abstract

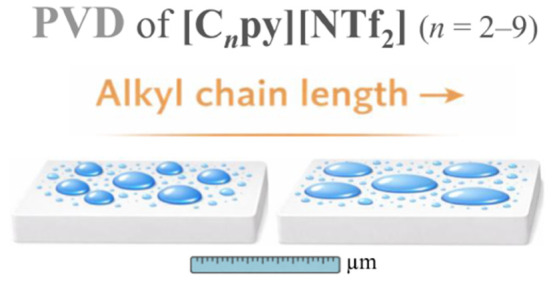

## Introduction

Ionic liquids (ILs) are a versatile class of compounds that have attracted increasing attention across chemistry, materials science, and engineering.^1–4^ Defined as salts composed entirely of cations and anions that melt at relatively low temperatures, ILs exhibit a unique combination of properties, including high thermal and chemical stability, negligible vapor pressure, high ionic conductivity, and wide electrochemical windows, making them attractive for a broad range of technological applications.^5–8^ Structurally, ILs consist of large, often asymmetric organic cations paired with organic or inorganic anions.^1,2^ By tuning the molecular features of these ions, their physicochemical properties can be precisely controlled, including density, viscosity, surface tension, and adhesion strength.^9–13^ Owing to their combined polar and nonpolar character, ILs exhibit a distinctive nanostructured organization arising from a balance between electrostatic interactions among ions and weaker van der Waals and π–π forces.^2,5,7^ The extent of this internal structuring is largely governed by the length and symmetry of the cation alkyl chains. Typically, the polar domains are formed by the charged cationic heads and the anions, while the aliphatic side chains aggregate to form nonpolar regions. Short alkyl chains lead to relatively small nonpolar domains, whereas increasing chain length enhances segregation between polar and nonpolar regions.^5,14^ Among the various IL families, imidazolium-based ILs have been the most extensively studied, owing to their stability and well-established structure–property relationships.^15–17^ In contrast, pyridinium-based ILs remain comparatively less explored, despite their rigid aromatic core, which may enhance π–π interactions and thermal stability.^18–21^ Pyridinium-based ILs have shown promising performance in CO_2_ capture,^22^ lubrication,^23^ and environmentally friendly applications, combining versatility with low toxicity and cost-effectiveness.^24^

Among the distinctive properties of ILs, their exceptionally low vapor pressure is particularly noteworthy, as it enables operation under high-vacuum conditions.^4^ This characteristic has opened new research directions focused on the preparation and behavior of thin IL films, as well as on their potential in emerging technologies.^25–33^ In this context, physical vapor deposition (PVD) has emerged as a powerful approach for fabricating IL films, offering excellent reproducibility and precise control over film nanostructure.^4^ These advantages have enabled detailed investigations of the mechanisms governing film formation.^25,27,30,32,34–36^ During deposition, ion pairs initially adsorb onto the substrate, preferentially occupying energetically favorable sites and initiating nucleation. The minimum free area for nucleation (MFAN), which is the smallest surface area able to sustain a stable cluster, is reduced by strong IL-substrate interactions, promoting more uniform nucleation. Depending on conditions such as temperature and deposition rate, ion pairs may also form small aggregates in the vapor phase before reaching the surface.^31^ Both isolated ion pairs and preformed clusters contribute to film growth, influencing nucleation dynamics, accelerating coalescence, and ultimately determining the film morphology. As deposition proceeds, adsorbed species merge through coalescence, which can be classified as first-order, involving primary clusters, or second-order, involving previously coalesced clusters (details of these mechanisms are presented in Figure S2 of the Supporting Information (SI)). The manner in which these clusters merge and spread across the surface is strongly governed by the adhesion between the IL and the substrate: strong adhesion favors layer-by-layer (two-dimensional) growth, whereas weaker adhesion promotes island-like (three-dimensional) growth. In some cases, ILs exhibit a combination of growth modes, where an initial continuous wetting layer forms before the material organizes into discrete three-dimensional domains.^35,37,38^ The wetting behavior of an IL on a substrate is a key factor influencing the mode of film growth and can be quantified by measuring the contact angle formed between a liquid droplet and the substrate surface. The contact angle (θ) reflects the balance of interfacial tensions among the solid–vapor (γ_s–v_), solid–liquid (γ_s–l_), and liquid–vapor (γ_l–v_) interfaces, as described by Young’s eq 1.^39^

1 $$\cos\theta =\frac{\gamma_{s-v}-\gamma_{s-l}}{\gamma_{l-v}}$$

The substrate plays a critical role in determining the contact angle, and its chemical composition, surface energy, and topography govern the interactions with the IL. As a result, the same IL can exhibit different contact angles on different substrates. For a given IL, the γ_l–v_ remains constant, so variations in contact angle primarily reflect differences in the substrate’s surface energy and the IL–substrate interfacial tension. According to Young’s equation, higher wettability is generally observed when the γ_s–v_ is high, while both γ_l–v_ and γ_s–l_ are relatively low.

Numerous studies have examined how thin-film formation of imidazolium-based ILs depends on factors such as substrate choice, deposition conditions, cation alkyl chain length, and even IL mixture composition.^25,27,32,35,36,40–42^ In contrast, systematic investigations of pyridinium-based IL films under comparable conditions remain scarce. Although pyridinium-based ILs share structural similarities with imidazolium systems, their distinct cation architecture may lead to different interfacial interactions and film morphologies. To address this gap, the present study systematically investigates how molecular structure influences the formation and morphology of pyridinium-based IL films on indium tin oxide (ITO) and gold (Au) substrates. The choice of ITO and Au was motivated not only by their widespread use in organic electronics and surface modification, but also by the opportunity to compare two substrates with significantly different surface energies, allowing us to probe the effect of substrate energetics on ionic liquid film morphology. A homologous series of ILs, [C_*n*_py]­[NTf_2_] (*n* = 2–9), was selected to enable a systematic evaluation of chain-length-dependent effects on film structure. The molecular structures of the corresponding ion pairs are shown in Figure 1a. By comparing films of ILs with identical deposited masses, this approach provides insight into how the combined effects of alkyl chain length and substrate interactions govern film morphology, wetting behavior, and nanostructural organization. The ILs were deposited by physical vapor deposition (PVD) using a modified Knudsen effusion setup, which provided a well-defined mass flow rate.^43^ As shown in Figure 1b, the ITO and Au substrates were mounted directly on the quartz crystal microbalance (QCM), ensuring that the registered mass closely corresponded to the amount of material deposited on each surface. Each IL was deposited simultaneously onto both substrates under identical conditions, ensuring reproducibility and direct comparability. Following deposition, the films were characterized in terms of morphology, using scanning electron microscopy (SEM) to investigate droplet shape and size distributions, and in terms of chemical composition, using X-ray photoelectron spectroscopy (XPS) and infrared spectroscopy (IR). This combined analysis provides insight into how alkyl chain length and substrate interactions govern film morphology, wetting behavior, and nanostructural organization, guiding the design of functional thin films for future applications. Such control is particularly relevant for applications where interfacial structure dictates performance, including organic and printed electronics, electrochemical devices, sensing platforms, lubrication coatings, energy storage interfaces, and surface functionalization.

**Figure 1:**
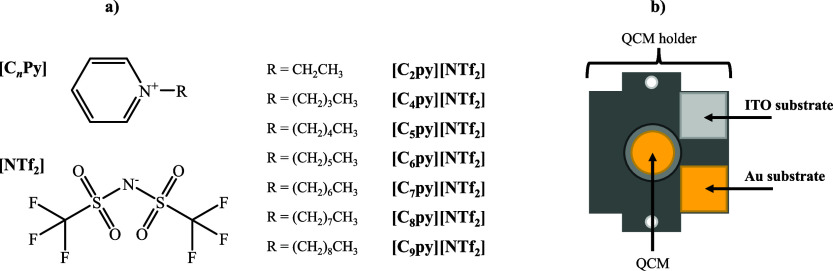
(a) Molecular structures of the studied ionic liquids: 1-ethylpyridinium bis­(trifluoromethylsulfonyl)­imide ([C_2_py]­[NTf_2_]); 1-butylpyridinium bis­(trifluoromethylsulfonyl)­imide ([C_4_py]­[NTf_2_]); 1-pentylpyridinium bis­(trifluoromethylsulfonyl)­imide ([C_5_py]­[NTf_2_]); 1-hexylpyridinium bis­(trifluoromethylsulfonyl)­imide ([C_6_py]­[NTf_2_]); 1-heptylpyridinium bis­(trifluoromethylsulfonyl)­imide ([C_7_py]­[NTf_2_]); 1-octylpyridinium bis­(trifluoromethylsulfonyl)­imide ([C_8_py]­[NTf_2_]); and 1-nonylpyridinium bis­(trifluoromethylsulfonyl)­imide ([C_9_py]­[NTf_2_]). (b) Schematic representation of the quartz crystal microbalance (QCM) holder used for the deposition of these ionic liquids, illustrating the position of the QCM sensor and the indium tin oxide (ITO) and gold (Au) substrates.

## Experimental Details

### Reagents

1-Ethylpyridinium bis­(trifluoromethylsulfonyl)­imide ([C_2_py]­[NTf_2_], CAS 712354-97-7), 1-butylpyridinium bis­(trifluoromethylsulfonyl)­imide ([C_4_py]­[NTf_2_], CAS 187863-42-9), 1-pentylpyridinium bis­(trifluoromethylsulfonyl)­imide ([C_5_py]­[NTf_2_]), 1-hexylpyridinium bis­(trifluoromethylsulfonyl)­imide ([C_6_py]­[NTf_2_], CAS 460983-97-5), 1-heptylpyridinium bis­(trifluoromethylsulfonyl)­imide ([C_7_py]­[NTf_2_]), 1-octylpyridinium bis­(trifluoromethylsulfonyl)­imide ([C_8_py]­[NTf_2_], CAS 384347-06-2), and 1-nonylpyridinium bis­(trifluoromethylsulfonyl)­imide ([C_9_py]­[NTf_2_]) were commercially obtained from IoLiTec with a stated purity of ≥99%. Prior to deposition, the ILs were placed in the vacuum system and exposed to elevated temperatures (373–423 K) to remove residual water and volatile impurities. This procedure also ensured that potential nonvolatile contaminants, such as polymeric derivatives present at ppm levels, did not interfere with film formation. The main properties of the ILs studied are summarized in the SI (Table S1).

### Substrates

Ionic liquids were deposited on indium tin oxide (ITO)-coated glass and gold (Au)-coated ITO/glass substrates to investigate the influence of the substrate on film growth. The ITO/glass substrates consisted of glass supports (10 × 10 × 1.1 mm) coated on one side with a 180 nm thick ITO layer and were commercially obtained from Praezisions Glas & Optik GmbH. All substrates were cleaned in an ultrasonic bath with high-purity ethanol prior to use. Au-coated substrates were prepared by depositing a 100 nm thick Au layer onto ITO/glass supports using a Cressington 108 Auto Sputter Coater operated in direct current (DC) magnetron mode, applying a discharge current of 30 mA for 60 s with a high-purity (≥99%) gold target. Prior to use, all substrates were stored in a desiccator to minimize exposure to air and potential contamination. Immediately before deposition, the substrates were transferred to a clean vacuum chamber (*p* < 10^–4^ Pa) and maintained under high-vacuum conditions. Ultrahigh-purity nitrogen was used to purge the chamber, further reducing the presence of residual gases. The surface topography of the substrates is presented in the SI (Figure S3).

### Thin Film Deposition

Thin films of pyridinium-based ILs, [C_*n*_py]­[NTf_2_], were deposited by vacuum thermal evaporation using a combined Knudsen effusion/quartz crystal microbalance (QCM) system developed in our laboratory.^43^ To prevent undesired chemical interactions between the ILs and the evaporation source, a stainless-steel Knudsen cell was employed. The Knudsen effusion cell allows fine control over the mass flow rate of the evaporating species, enabling the formation of well-defined films according to the volatility of each IL. During deposition, the QCM resonance frequency was continuously recorded, and its temporal variation (*df*/*dt*), expressed in Hz·s^–1^, was used to determine the mass deposition flux (φ_*m*_). The flux was calculated using the Sauerbrey equation, assuming a linear response of the QCM frequency to mass loading. This assumption is justified because, although ILs may deviate from Sauerbrey behavior at higher coverages, only small amounts were deposited in the present experiments, and the IL remained confined to the crystal surface, resulting in rigid coupling and negligible viscoelastic effects. The mass deposition flux is related to the measured frequency change by eq 2, where *S*_q_ is the theoretical mass sensitivity coefficient. For the 6 MHz AT-cut quartz crystal resonator used in this work, *S*_q_ = −0.0815 Hz·ng^–1^·cm^2^ at *T* = 298 K.^43^ For reporting purposes, φ_*m*_ was converted into the deposition rate (φ), expressed in Å·s^–1^, using the material density (ρ), expressed in g·cm^–3^. The total mass deposited per unit area (*m*), expressed in μg·cm^–2^, was obtained by integrating the frequency shift over the deposition time according to eq 3, where Δ*f* is the total change in the QCM resonance frequency during deposition.

2 $$\varphi =10^{-1}\times \frac{df/dt}{\rho \times S_{q}}$$

3 $$m=10^{-3}\times \frac{1}{S_{q}}\times \Delta f$$

All depositions were carried out on two different substrates, ITO/glass and Au/ITO/glass, to investigate the influence of surface properties on film growth. The substrate temperature was maintained at *T* = 278 K throughout the experiments. Previous studies have shown that ILs generally exhibit stronger adhesion and higher wettability on Au surfaces, whereas adhesion is weaker on ITO.^25,32,36^ Moreover, the wettability and resulting film morphology can depend on the alkyl chain length of the cation, making these two substrates particularly suitable for studying chain-length-dependent effects.^25,36^ The substrates were fixed onto the QCM support (Figure 1b) using carbon adhesive tape. This mounting strategy ensures that the mass registered by the QCM corresponds directly to the amount of material condensed onto the substrates. In this configuration, the QCM and substrates are vertically aligned with the effusion cell, minimizing positional uncertainty and enabling accurate measurements of both mass flow and deposited material. In this study, we first investigated how the value of φ influences the morphology of [C_4_py]­[NTf_2_] films on both ITO and Au/ITO substrates. φ was controlled by adjusting the vaporization temperature of the IL. Films were produced at two deposited masses, *m* = 7.3 ± 0.5 μg·cm^–2^ (≈50 nm) and *m* = 14.6 ± 0.5 μg·cm^–2^ (≈100 nm). Thin films of [C_4_py]­[NTf_2_] were prepared at φ of 0.1, 0.3, 0.5, and 1.1 Å/s, corresponding to effusion cell temperatures of *T* = 483.15, 493.15, 523.15, and 533.15 K, respectively. Following the initial investigation of [C_4_py]­[NTf_2_], thin films of the pyridinium-based IL series, [C_2_py]­[NTf_2_] to [C_9_py]­[NTf_2_], were deposited on both ITO and Au/ITO substrates. All ILs in the series were deposited at a deposition rate close to 0.3 Å/s, with effusion cell temperatures of *T* = 503.15 K for [C_2_py]­[NTf_2_] and [C_4_py]­[NTf_2_], and *T* = 508.15 K for [C_5_py]­[NTf_2_], [C_6_py]­[NTf_2_], [C_7_py]­[NTf_2_], [C_8_py]­[NTf_2_], and [C_9_py]­[NTf_2_]. The same two deposited masses were used for all ILs, allowing a direct comparison of the effect of alkyl chain length on film morphology and wetting behavior. The specific deposition conditions are summarized in the SI (Tables S3 and S4).

## Characterization Methods

### Scanning Electron Microscopy (SEM)

The surface morphology of the IL films was characterized by scanning electron microscopy (SEM, Hitachi, FlexSEM 1000). Images were recorded at multiple magnifications (650×, 1600×, and 4000×) to evaluate the substrate features as well as the distribution and size of micro- and nanodroplets, or the formation of coalesced films, depending on the deposition conditions and substrate type. Higher-resolution micrographs were acquired using a backscattered (BSE) detector. An accelerating voltage of 3 kV was employed for samples on ITO, while 5 kV was used for films deposited on Au/ITO. An in-lens detector was operated with a working distance of approximately 7 mm. All imaging conditions were optimized to limit possible beam-induced alterations to the IL films. Subsequent quantitative and qualitative analysis of the SEM micrographs was carried out using the *ImageJ* software package.^44^ In addition to SEM, the surface morphology of the IL films was also examined by optical microscopy to provide complementary information on film coverage and the distribution of droplets at larger scales.

### X-ray Photoelectron Spectroscopy (XPS)

X-ray photoelectron spectroscopy (XPS) analyses were carried out using an ESCALAB 250 Ci spectrometer (Thermo Scientific) equipped with a monochromatic Al–Kα radiation source (1486.6 eV) operated at 200 W. The X-ray beam had a spot size of approximately 650 μm on the sample surface. Spectra were collected at a takeoff angle of 90° relative to the sample surface using a hemispherical electron energy analyzer operating in constant analyzer energy (CAE) mode. A pass energy of 150 eV with an energy step of 1 eV was employed for survey scans, while high-resolution spectra were acquired with a pass energy of 40 eV and a step size of 0.1 eV. High-resolution spectra were fitted using Gaussian–Lorentzian peak shapes and a Shirley-type background. Detailed spectra were recorded for the C 1s, O 1s, N 1s, F 1s, S 2p, In 3d, Sn 3d, and Au 4f core levels. Data processing and quantification were performed using CasaXPS software (version 2.3),^45^ with relative sensitivity factors obtained from the Kratos library.

### Reflectance Infrared Spectroscopy

Reflectance infrared spectroscopy of the IL films was performed in the mid-infrared range (600–4000 cm^–1^) using a Bruker Vertex 80v FT-IR spectrometer operating in reflectance-absorption mode. Spectra were collected at normal incidence with the samples mounted on the stage of a Hyperion microscope coupled to the spectrometer. A clean substrate without deposited material was used to acquire the background spectrum. All measurements were carried out with a spectral resolution of 4.0 cm^–1^, and each spectrum represents the average of 64 accumulated scans. Attenuated total reflection Fourier-transform infrared (ATR-FTIR) measurements of the bulk ionic liquids were obtained using a Spectrum Two FT-IR spectrometer (PerkinElmer, USA) equipped with a diamond attenuated total reflectance crystal (GladiATR Accessory S2PE, PIKE Technologies, USA). Spectra were recorded in the 4000–550 cm^–1^ wavenumber range using the PerkinElmer Spectrum software. Each spectrum was collected by averaging 32 scans at a resolution of 4 cm^–1^.

## Results and Discussion

Initially, a systematic study of the evaporation temperature and the resulting deposition flow rate was conducted to assess their impact on the morphology of IL films. Figure 2 presents SEM images of [C_4_py]­[NTf_2_] films deposited on ITO/glass for two different deposited amounts: *m* = 7.3 ± 0.5 μg·cm^–2^ (Figure 2a–d), deposited at φ = 0.1–1.1 Å/s, and *m* = 14.6 ± 0.5 μg·cm^–2^ (Figure 2e–h), deposited at φ = 0.1–1.2 Å/s. The deposition flow rate was varied by increasing the evaporation temperature from 483 K to 533 K. The corresponding droplet size-distribution histograms, derived from these SEM images, are shown in Figure 2i–l for the *m* = 7.3 μg·cm^–2^ films and Figure 2m–p for the 14.6 μg·cm^–2^ films. Optical microscopy images of the same samples, along with the results obtained on the Au substrate, are presented in the SI (Figures S4 and S5). These data enable a quantitative evaluation of the micro- and nanodroplet populations formed under the various deposition conditions. On the ITO substrate, the effect of the flow rate on film morphology is clearly evident (Figure 2 and Figure S4), with this influence being particularly pronounced for the 14.6 μg·cm^–2^ samples. Microdroplet formation is observed at all investigated flow rates. Increasing the φ leads to larger droplets and more pronounced coalescence, as evidenced by the observed increase in droplet size, indicating enhanced growth dynamics. A similar trend is observed for the 7.3 μg·cm^–2^ films; however, the lower deposited mass limits the extent of coalescence, resulting in overall smaller droplets. These observations are consistent with previous studies on imidazolium-based ILs, in which higher deposition rates – achieved by increasing the evaporation temperature – were also shown to enhance droplet coalescence.^42^ The size distribution histograms further quantify these morphological changes.

**Figure 2:**
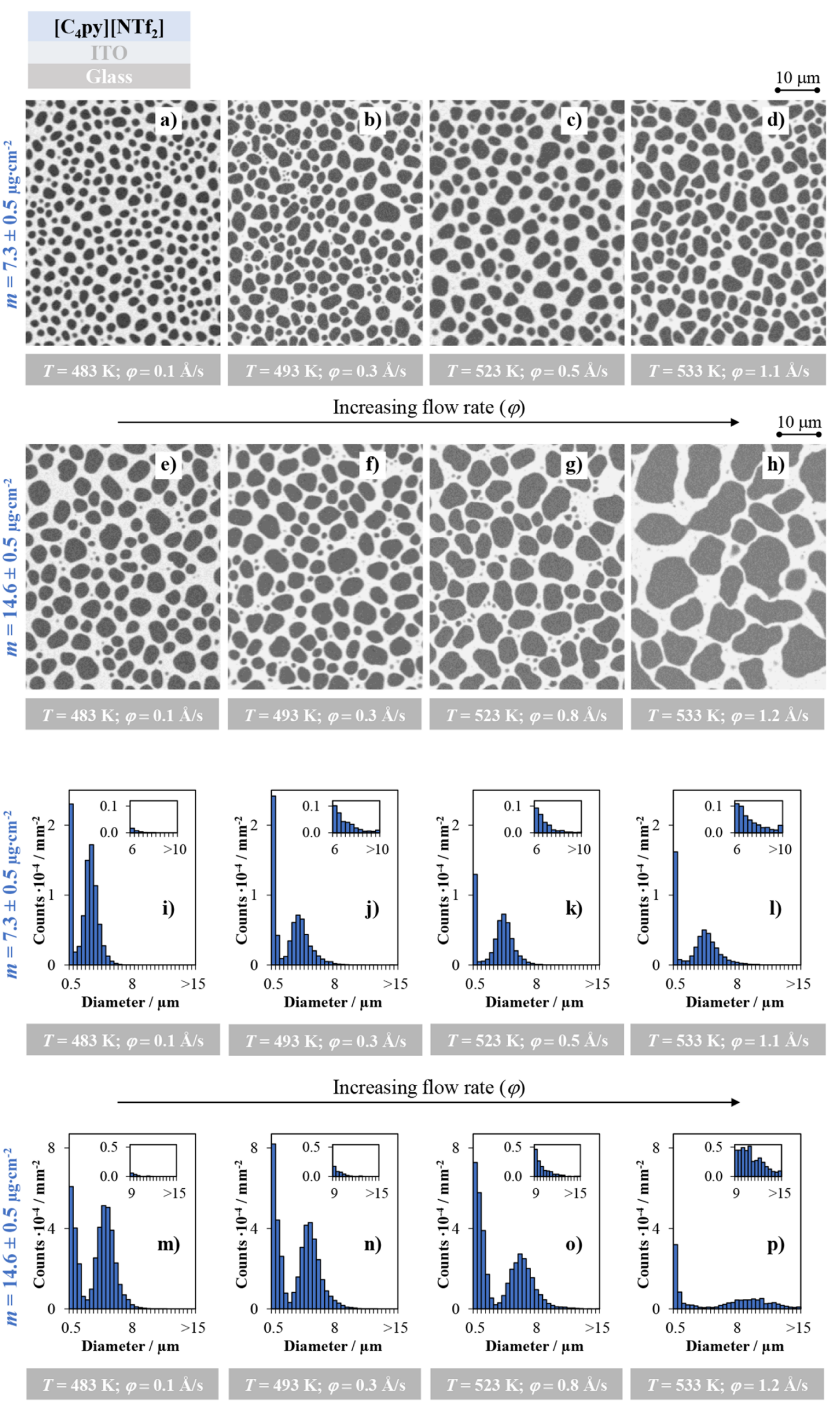
SEM images (a–h) and droplet size distributions (i–p) of [C_4_py]­[NTf_2_] thin films deposited on ITO-coated glass substrates at different evaporation temperatures/different flow rates (φ). Experimental data are shown for deposition amounts of 7.3 ± 0.5 μg·cm^–2^ (thickness of ≈50 nm, micrographs a, b, c, and d and histograms i, j, k, and l) and 14.6 ± 0.5 μg·cm^–2^ (thickness of ≈100 nm, micrographs e, f, g, and h and histograms m, n, o, and p). Top-view images were acquired using a backscattered electron detector.

For the 7.3 μg·cm^–2^ films (Figure 2 i–l), increasing the φ from 0.1 to 1.1 Å/s leads to a progressive shift of the droplet size distribution toward larger diameters, accompanied by an increase in the modal droplet size. Modal diameters of 2.8 μm, 3.0 μm, 4.0 μm, and 4.0 μm are obtained at φ = 0.1 Å/s (Figure 2i), 0.3 Å/s (Figure 2j), 0.5 Å/s (Figure 2k), and 1.1 Å/s (Figure 2l), respectively. Although the modal diameter remains unchanged between 0.5 Å/s and 1.1 Å/s, a closer inspection of Figure 2l reveals a significantly higher population of droplets with diameters exceeding 10 μm, a feature not observed in Figure 2k. This indicates that coalescence is more extensive at φ = 1.1 Å/s despite the identical modal size. For the 14.6 μg·cm^–2^ films (Figure 2m–p), the influence of the flow rate is even more pronounced. The larger amount of material available during growth significantly enhances coalescence, leading to both broader droplet size distributions and a systematic increase in the modal droplet diameter. Modal diameters of 4.9 μm at φ = 0.1 Å/s (Figure 2m), 4.9 μm at φ = 0.3 Å/s (Figure 2n), 5.9 μm at φ = 0.8 Å/s (Figure 2o), and 9.9 μm at φ = 1.2 Å/s (Figure 2p) are observed. These results demonstrate that thicker films readily evolve into larger droplets even at relatively low deposition rates, and that the impact of deposition rate becomes increasingly significant as the total deposited mass increases. Figure S5 of the SI shows the results obtained on the Au surface. On Au, there is extensive spreading compared to ITO; however, it is also noteworthy that microdroplets grow and coalesce significantly when the flow rate is increased from 0.1 to 0.3 Å/s. The enhanced coalescence observed at higher φ can be rationalized by considering contributions from both surface dynamics and vapor-phase processes. At higher source temperatures, which lead to increased deposition rates, deposited ion pairs arrive at the substrate with higher kinetic energy, enhancing their surface mobility. This increased mobility promotes droplet–droplet interactions and merging events, consistent with Ostwald ripening, where larger droplets grow at the expense of smaller ones due to surface diffusion.^46^ In addition, as highlighted by Matsumoto and Maruyama, the assumption that ion pairs always reach the substrate as isolated entities may not hold under all deposition conditions.^31^ At elevated deposition rates, partial association in the vapor phase becomes more likely, leading to the formation of small ion-pair aggregates or cluster-like species. Upon arrival at the surface, these larger building units can further accelerate droplet growth by enhancing coalescence and increasing the effective mass per nucleation event. Importantly, the morphology of the IL films was found to be stable over time when stored under appropriate conditions. This stability is illustrated in Figure 3, which presents optical microscopy images of [C_4_py]­[NTf_2_] films.

**Figure 3:**
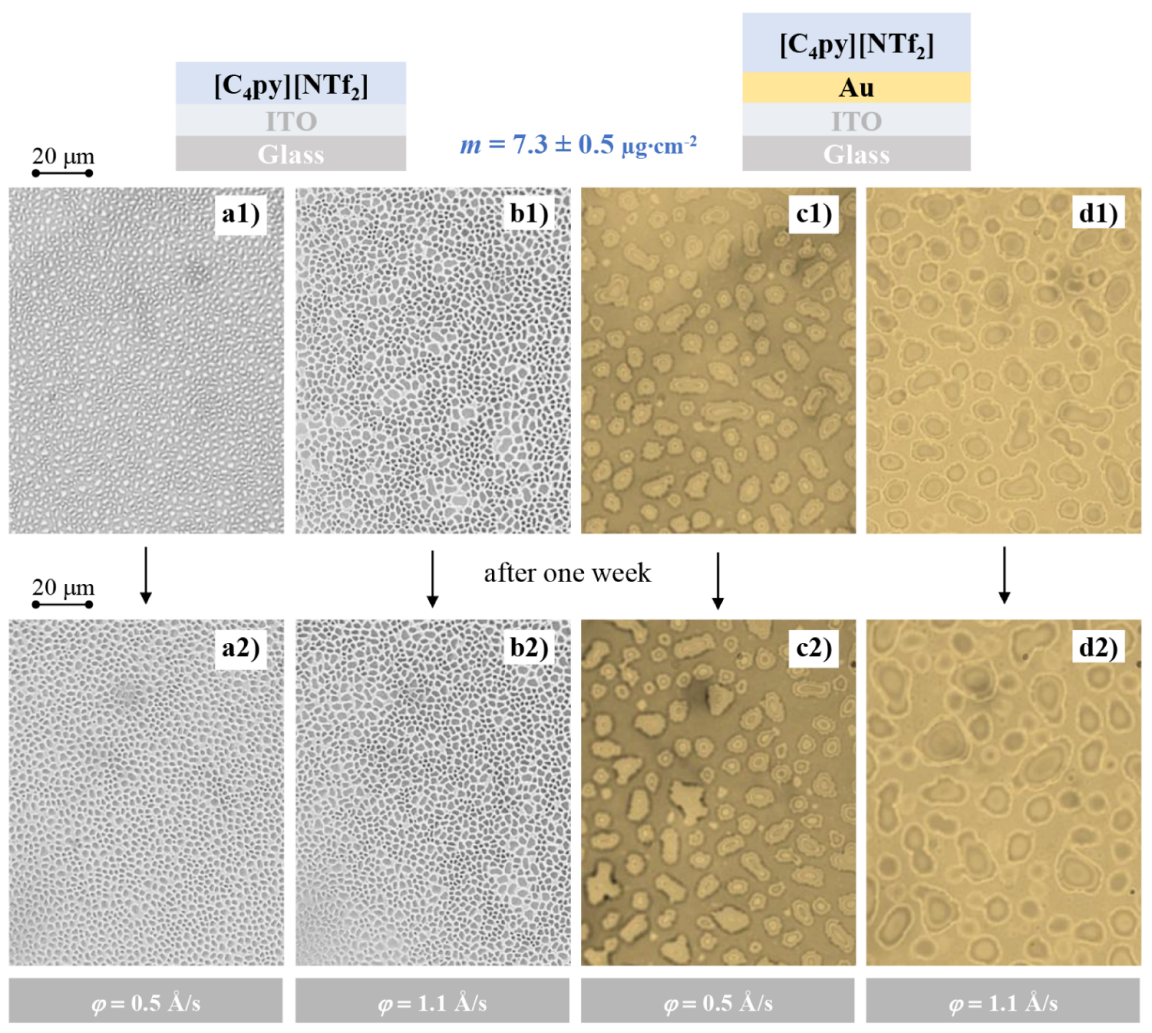
Optical microscopy images of [C_4_py]­[NTf_2_] thin films deposited at a deposition amount of 7.3 ± 0.5 μg·cm^–2^ using two flow rates (φ = 0.5 and 1.1 Å·s^–1^) on ITO-coated glass (a1, a2, b1, b2) and Au/ITO-coated glass (c1, c2, d1, d2) substrates. Samples were visualized immediately after deposition (a1–d1) and 1 week later (a2–d2).

These films were deposited at two different φ on both ITO (a1, a2, b1, b2) and Au/ITO substrates (c1, c2, d1, d2). Images a1–d1 were acquired immediately after deposition. It should be noted that the optical microscope observations were performed ex situ at room temperature after removal from the vacuum chamber. While ultrafast morphological changes immediately after deposition cannot be entirely excluded, the droplet morphologies remained largely consistent over time, as illustrated by images a2–d2 recorded 1 week later, indicating that the observed trends reliably reflect the intrinsic behavior of the films. The high degree of similarity between the corresponding image pairs indicates the absence of significant coalescence, restructuring, or degradation during storage. This observation is consistent with our previous studies, where SEM analysis of IL films after several days showed unchanged droplet size distribution and morphology.^32^ The scope of the present study was to investigate general trends in film morphology and wettability under standard laboratory conditions. Stability under extreme conditions, such as high temperature, high humidity, or device-relevant environments, could be explored in future work. Building on the insights gained from the flow-rate study, the analysis was extended to the full pyridinium-based IL series, with all films deposited at a similar flow rate (φ = 0.3 ± 0.1 Å·s^–1^) to assess the effect of alkyl chain length on film morphology. A moderate deposition rate was chosen, as higher rates would lead to rapid droplet coalescence, obscuring the effects of alkyl-chain length and substrate surface energy. Figure 4 presents SEM micrographs of IL films containing cations with different alkyl chain lengths. Optical microscopy images are presented in the SI (Figure S6). Films were deposited on ITO at two nominal deposited amounts, *m* = 7.3 ± 0.5 μg·cm^–2^ (≈50 nm, micrographs a1–g1) and *m* = 14.6 ± 0.5 μg·cm^–2^ (≈100 nm, micrographs a2–g2). The corresponding films grown on Au/ITO are also shown (micrographs h1–n1 and h2–n2). On ITO/glass, examination of the 14.6 μg·cm^–2^ films (micrographs a2–g2) reveals a clear dependence of the morphology on the cation alkyl chain length. As the alkyl chain length increases, the films exhibit progressively larger droplets with increasingly irregular shapes. ILs with shorter alkyl chains form smaller, more spherical nanodroplets, whereas long-chain ILs produce larger, more laterally spread droplets, consistent with lower contact angles and increased wetting of the substrate.

**Figure 4:**
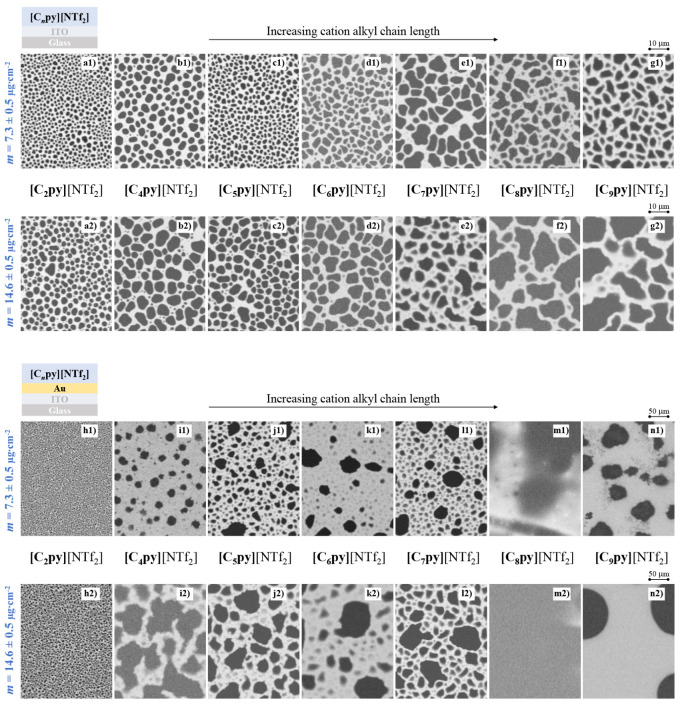
Morphology of [C*_n_* py]­[NTf_2_] thin films deposited on ITO-coated glass (a–g) and Au/ITO-coated glass (h–n) substrates. Shown are films of [C_2_py]­[NTf_2_] (a1, a2, h1, h2), [C_4_py]­[NTf_2_] (b1, b2, i1, i2), [C_5_py]­[NTf_2_] (c1, c2, j1, j2), [C_6_py]­[NTf_2_] (d1, d2, k1, k2), [C_7_py]­[NTf_2_] (e1, e2, l1, l2), [C_8_py]­[NTf_2_] (f1, f2, m1, m2), and [C_9_py]­[NTf_2_] (g1, g2, n1, n2). The data correspond to deposition amounts of 7.3 ± 0.5 μg·cm^–2^ (micrographs a1–g1 and h1–n1) and 14.6 ± 0.5 μg·cm^–2^ (micrographs a2–g2 and h2–n2), obtained at a constant deposition rate of φ ≈ 0.3 Å·s^–1^. Top-view SEM images were acquired using a backscattered electron detector. Corresponding optical microscopy images are presented in the SI (Figure S6).

This behavior is attributed to enhanced coalescence processes associated with longer alkyl chains. A comparable trend is observed for the 7.3 μg·cm^–2^ films (micrographs a1–g1), where the droplet size systematically increases with alkyl chain length. However, owing to the smaller amount of deposited material, coalescence is less extensive than in thicker films. For the 14.6 μg·cm^–2^ films deposited on Au/ITO (micrographs h2–n2), the influence of alkyl chain length is even more pronounced. Increasing the chain length leads to markedly enhanced coalescence and improved substrate wettability. In particular, the thicker [C_8_py]­[NTf_2_] and [C_9_py]­[NTf_2_] films (micrographs m2 and n2) exhibit a morphology distinct from that of the shorter-chain ILs, forming highly coalesced films that spread more extensively over the Au surface, suggesting a strong interfacial affinity and reduced interfacial tension. This distinct wetting behavior can be rationalized by considering the strong adsorption of the first monolayers of long-chain ILs on Au. Such adsorption promotes enhanced spreading of subsequent layers through dispersive interactions between the alkyl chains, favoring the layer-by-layer growth.^30^ Additional insight is provided by Young’s eq (eq 1). For a given IL, the liquid–vapor surface tension (γ_l–v_) remains constant; therefore, variations in the contact angle arise from differences in the solid–vapor surface energy (γ_s–v_) and the solid–liquid interfacial tension (γ_s–l_). Au exhibits a significantly higher γ_s–v_ (1.25 J·m^–2^)^47^ compared to ITO (0.0291 J·m^–2^),^48^ which accounts for the lower contact angles and enhanced spreading observed on Au. In contrast, the much lower γ_s–v_ of ITO contributes to poorer wetting, resulting in more spherical droplets with higher contact angles. Substrate roughness is nearly identical for both ITO and Au/ITO substrates (Rq ≈ 4 nm), suggesting that differences in droplet morphology are dominated by surface energy considerations rather than topography (Figure S3). A similar interpretation applies to both 7.3 μg·cm^–2^ and 14.6 μg·cm^–2^ films. In the case of thinner films, although the general tendency toward larger droplets and lower contact angles persists, the reduced amount of deposited material prevents the formation of fully coalesced films. Instead, large microstructures with low contact angles are observed alongside uncovered regions of the substrate. For ILs with shorter alkyl chains, droplets are still present but are larger and exhibit lower contact angles on Au than on ITO, further confirming the enhanced wetting behavior on Au. Previous studies on imidazolium-based ILs have been performed under comparable conditions, and the trends observed in film formation on both Au and ITO substrates were very similar to those reported here for pyridinium-based ILs.^25,27,36^ The wetting behavior of ionic liquids on solid substrates has been extensively investigated, highlighting the role of alkyl chain length and substrate surface energy in controlling droplet formation and spreading.^8,25,31,33,49–55^ Contact angles of approximately 30° were reported for droplets of various imidazolium-based ILs on glass surfaces, whereas the same ILs deposited on Au surfaces exhibited predicted contact angles below 10°.^55^ These observations are consistent with the trends seen in our previous studies and in the present work, where IL spreading is more pronounced on Au than on ITO.^25,36,40^ Other studies reported contact angles of approximately 14° for nanodroplets of imidazolium-based ILs with different anions ([NTf_2_] and [PF_6_]) deposited on ITO substrates.^31^ In a recent study from our group, microdroplets of short-chain imidazolium-based ILs vapor-deposited on ITO substrates were imaged by AFM, and contact angles of θ = 20 ± 4° were determined.^25^ It should be noted that nanoscale imaging of IL nanodroplets is experimentally challenging, as tip–sample interactions during AFM measurements can induce droplet deformation or coalescence, potentially altering the intrinsic morphology. Although AFM characterization was not performed for the pyridinium-based ILs in the present study, the comparable droplet morphology and sphericity observed suggest that these microdroplets likely exhibit contact angles similar to those reported for imidazolium-based congeners. To complement the analysis, the SEM images of the IL films deposited on ITO were carefully and systematically processed using *ImageJ* to extract quantitative information on droplet density and modal diameter, considering only droplets larger than 1 μm. Figure 5 summarizes these results. The red curve shows the droplet density (counts per mm^2^), and the black curve represents the modal droplet diameter, both plotted as a function of the cation alkyl chain length. The results indicate that ILs with longer alkyl chains, particularly [C_8_py]­[NTf_2_] and [C_9_py]­[NTf_2_], display behavior significantly different from their shorter-chain counterparts. These long-chain ILs exhibit much lower droplet densities and considerably larger modal diameters, reflecting enhanced coalescence and spreading on the ITO surface. This observation aligns with the SEM analyses described previously, where [C_8_py]­[NTf_2_] and [C_9_py]­[NTf_2_] films formed more continuous structures with larger and irregular droplets. The enhanced spreading can be attributed to the lower γ_l–v_ characteristic of ILs with longer alkyl chains.^56^ Additionally, increased chain length strengthens lateral interactions among alkyl chains, promoting droplet merging and the formation of larger aggregates. A notable odd–even effect is also evident across the series. ILs with even-numbered alkyl chains tend to form droplets with larger modal diameters and correspondingly lower droplet densities, whereas odd-numbered chains produce smaller droplets.

**Figure 5:**
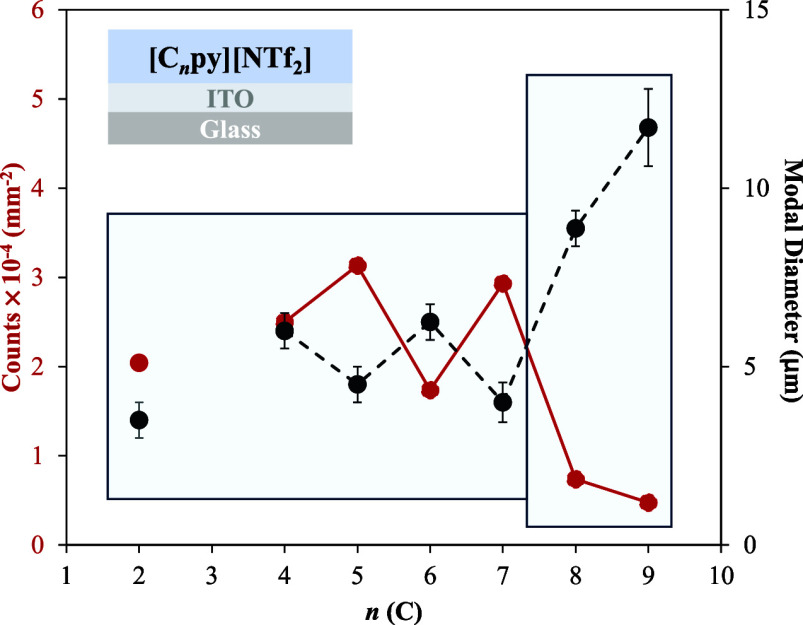
Droplet count (red) and modal diameter (black) as a function of the number of carbons in the alkyl chain for droplets with diameters greater than 1 μm. Data correspond to films with a deposition amount of 14.6 ± 0.5 μg·cm^–2^.

This trend indicates that subtle differences in molecular packing and chain conformation, depending on the parity of the alkyl chain, influence the organization of IL molecules at the interface and the subsequent evolution of the film during deposition. A possible explanation for this odd–even effect is that ILs with even-numbered alkyl chains adopt more extended and symmetric conformations, which enable more efficient packing at the interface. This improved packing strengthens van der Waals interactions between neighboring chains, promoting the formation of larger droplets and a lower overall droplet density, as illustrated in Figure 5. In contrast, odd-numbered chains are more likely to adopt less favorable conformations, resulting in decreased packing efficiency and increased interfacial energy. The resulting structural mismatch may limit droplet coalescence, resulting in a higher number of smaller droplets. Additional support for the odd–even effect is provided by a detailed analysis of the temperature-dependent density and viscosity of these ILs. Specifically, the thermal expansion coefficient (α), obtained from density measurements, and the Vogel temperature (*T*_0_), derived from viscosity data, were analyzed. Detailed results are provided in the SI (Table S2, Figure S1). A clear odd–even effect is observed: the odd-numbered ILs ([C_3_py]­[NTf_2_] and [C_5_py]­[NTf_2_]) display *T*_0_ values systematically higher than expected based on the linear fit of the even-numbered series ([C_4_py]­[NTf_2_] and [C_6_py]­[NTf_2_]), indicating lower molecular mobility. Furthermore, α is systematically higher for the odd-numbered ILs. Accordingly, as demonstrated in this work, the droplet size distribution of the odd-numbered ILs deviates from the trend defined by the even-numbered series. The reduced mobility of the odd-numbered ILs likely limits surface diffusion and secondary coalescence, leading to smaller microdroplets. In contrast, even-numbered ILs retain higher mobility, promoting more extensive droplet fusion and resulting in larger droplets. Additionally, the α also displays a clear odd–even alternation, further supporting the existence of systematic structural differences between even- and odd-numbered chains. Although α does not directly quantify molecular diffusion, its parity trend is consistent with the distinct packing and intermolecular organization underlying the observed morphological behavior. Odd–even effects have been widely documented for various properties in different classes of homologous compounds, where they influence multiple thermodynamic and physical characteristics.^57–61^ These effects have been shown to markedly influence the structure and morphology of thin films and self-assembled monolayers on solid substrates, arising from subtle variations in molecular packing and interfacial interactions.^62–65^ Notably, this specific odd–even effect has not been previously reported for IL films. These structural variations can modulate interfacial tension and wetting behavior, ultimately affecting nucleation, growth, and coalescence during film formation. Previous studies have reported a pronounced change in wetting and morphological behavior for ILs bearing alkyl chains with six or more carbon atoms. Beyond this threshold, the films exhibit a distinct morphological evolution, suggesting that longer alkyl chains promote altered interfacial organization and enhanced coalescence compared to their shorter-chain counterparts.^27,36^

Having established the morphological trends as a function of deposition conditions, alkyl chain length, and substrate, the chemical structure of the resulting IL films was subsequently investigated. Figure 6 presents the infrared absorption spectra of some [C_*n*_py]­[NTf_2_] ILs (*n* = 2, 8, and 9), comparing the bulk materials with the corresponding thin films and examining the influence of alkyl chain length and substrate. The detailed FTIR spectra are provided in the SI (Figures S7–S12). Overall, the spectra demonstrate that the ILs maintain their chemical integrity upon film formation.^66^ A comparison between the spectra of the bulk ILs and the thin films (Figure 6a–c) shows that both forms exhibit essentially the same set of vibrational bands in the 900–1400 cm^–1^ region. This spectral range corresponds predominantly to internal vibrational modes of the [NTf_2_]^−^ anion, which dominates the mid-infrared response of these [C_*n*_py]­[NTf_2_] ILs. The band near 1350 cm^–1^ (1350, 1348, and 1348 cm^–1^ for *n* = 2, *n* = 8, and *n* = 9, respectively) corresponds to the ν_as_(SO_2_) asymmetric stretching mode. The bands centered around 1200 cm^–1^ (1185, 1188, and 1194 cm^–1^ for *n* = 2, *n* = 8, and *n* = 9, respectively) and 1135 cm^–1^ arise from the overlap of several C–F and S–O stretching vibrations, including ν_s_(CF_3_), ν_as_(CF_3_), and coupled modes of the [NTf_2_]^−^ anion. The band located near 1050 cm^–1^ (1055, 1057, and 1057 cm^–1^ for *n* = 2, *n* = 8, and *n* = 9, respectively) is assigned to the ν_as_(S–N–S) asymmetric stretching mode of the anion. These assignments are fully consistent with literature reports for ILs containing the [NTf_2_]^−^ anion.^67,68^ The preservation of these characteristic bands, without the appearance of new features or loss of existing ones, indicates that the core ionic structure remains intact upon transition from the bulk IL to the thin film. The influence of the substrate is evaluated in Figure 6d, where films deposited on ITO/glass and Au/ITO/glass are compared. The spectra remain highly similar, further confirming that the IL structure is preserved regardless of the underlying substrate. Subtle differences in relative band intensities are consistent with variations in film morphology or molecular orientation induced by differences in surface energy, as commonly observed for surface-confined IL layers. Importantly, these variations do not alter the vibrational signature of the anion, confirming the absence of substrate-induced chemical changes. Figure 6e compares the bulk ATR-FTIR spectra of [C_2_py]­[NTf_2_], [C_8_py]­[NTf_2_], and [C_9_py]­[NTf_2_]. As expected, all samples exhibit essentially the same vibrational pattern dominated by the [NTf_2_]^−^ anion. Subtle differences are observed with increasing chain length, with the band near 1200 cm^–1^ exhibiting a more pronounced shift in peak position across the three ILs (*n* = 2, 8, and 9; see insets of Figure 6e and f), while its intensity remains essentially unchanged.

**Figure 6:**
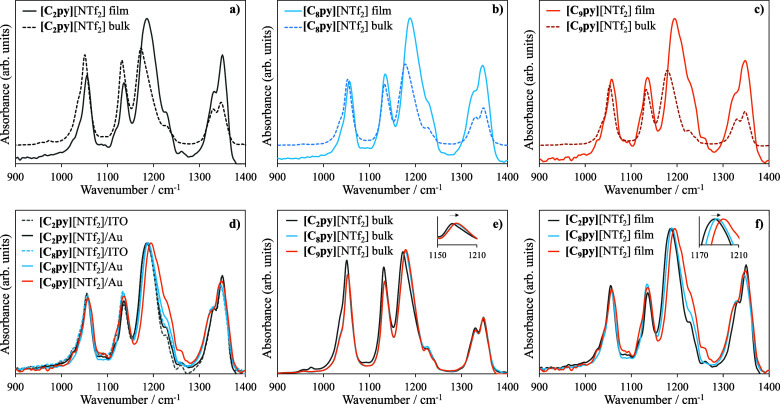
Infrared absorption spectra of [C*_n_* py]­[NTf_2_] ILs in bulk and as thin films. (a–c) Comparison between thin film spectra and bulk spectra of [C_2_py]­[NTf_2_], [C_8_py]­[NTf_2_], and [C_9_py]­[NTf_2_], respectively. (d) Comparison between films deposited on ITO/glass and Au/ITO/glass substrates. (e) Comparison of bulk spectra of [C_2_py]­[NTf_2_], [C_8_py]­[NTf_2_], and [C_9_py]­[NTf_2_]. (f) Comparison of spectra of thin films deposited on Au/ITO/glass. Thin films correspond to a deposition amount of 14.6 ± 0.5 μg·cm^–2^, and the spectra were obtained by specular reflectance using a spectrometer operating in reflectance-absorption mode. Bulk spectra were recorded in ATR mode by placing a drop of the IL directly on the ATR crystal.

This shift likely arises from stronger cation–anion interactions in the polar domain, promoted by the elongation of the nonpolar alkyl chains, which enhances segregation between polar and nonpolar regions.^69^ Finally, the thin film spectra of the three ILs (Figure 6f) exhibit the same internal consistency observed in the bulk samples. Small shifts in the region around 1200 cm^–1^, already present in the bulk spectra, are even more pronounced in the thin films, particularly for [C_9_py]­[NTf_2_]. While the precise origin of the more pronounced peak shift in thin films compared to bulk is not fully understood, it may be related to the stronger influence of the interfacial environment in thin films. In particular, confinement at the substrate surface and enhanced segregation between polar and nonpolar domains could slightly alter the vibrational environment of the anion.

XPS was employed to assess the surface composition and integrity of the IL film surfaces. The overall XPS results are provided in the SI (Figures S13–S18). Prior to XPS measurements, all samples were stored under an argon atmosphere to limit surface contamination. Nevertheless, the complete removal of carbonaceous species is notoriously difficult, as these substrates intrinsically carry adventitious carbon. Moreover, the XPS measurement itself may contribute additional contamination. Analysis of the bare substrate (Figure S13) indicates the presence of adventitious carbon, where carbon-related species account for approximately 30–40% of the detected surface signal. The corresponding C 1s spectra display characteristic C–C, C–O, and C=O contributions. The thickness of this layer is estimated to be no more than 1–2 nm. The XPS survey spectrum of ITO/glass following argon-ion sputtering, which effectively removes adventitious carbon, is shown in Figure S14. Although such sputter cleaning was not applied in the present deposition experiments, exposure of the substrates to ambient air was consistently minimized. It is important to note that surface contamination can significantly affect the growth behavior of IL films. At the nanoscale, investigations ideally require well-defined and clean solid surfaces, since even small amounts of carbon can alter nucleation and the formation of the first monolayers. For example, Deyko et al. reported that vapor-deposited [C_1_mim]­[NTf_2_] grows as three-dimensional islands from the outset on clean surface regions, whereas on carbon-contaminated areas it initially forms two-dimensional wetting layers before the onset of three-dimensional island growth.^70^ In this work, however, the emphasis is placed on relatively thick IL films, rather than on initial monolayer adsorption, where the influence of adventitious carbon is most pronounced. At the mesoscale, we have recently shown that the extent of morphological changes in IL films is influenced by both the thickness of the carbon layer on the substrate surface and the amount of deposited IL.^40^ These effects are particularly pronounced at low IL coverages and high levels of surface carbon, whereas for thicker IL films and substrates containing only the typical adventitious carbon, their influence on IL morphology is considerably reduced. While some degree of surface contamination is unavoidable and should be acknowledged, substrates are commonly employed in this condition for practical applications. Therefore, the primary objective is to reduce contamination to the greatest extent possible in order to limit its impact on the interpretation of the experimental results. Figure 7 presents high-resolution XPS spectra of [C_2_py]­[NTf_2_] and [C_9_py]­[NTf_2_] thin films deposited with an equivalent amount of 14.6 ± 0.5 μg·cm^–2^ on ITO/glass substrates (a–f) and Au/ITO/glass substrates (g–l). The XPS survey spectra are presented in Figures S15–S18 of the SI. A pronounced dependence on alkyl chain length is evident in the attenuation of substrate-related core levels. For [C_2_py]­[NTf_2_], the In 3d (centered at 444.3 and 451.7 eV) and Au 4f peaks (centered at 83.9 and 87.6 eV) remain clearly visible, indicating that a significant fraction of the substrate surface remains accessible to XPS detection. In contrast, these signals are strongly attenuated for [C_9_py]­[NTf_2_], despite the identical deposited mass, demonstrating more effective surface coverage. This behavior is consistent with a growth mode in which [C_2_py]­[NTf_2_] forms small droplets or islands, leaving exposed regions of the substrate, whereas the longer alkyl chain in [C_9_py]­[NTf_2_] enhances wetting and lateral spreading, resulting in a more continuous film, in agreement with the SEM observations shown in Figure 4. The C 1s spectra of both ILs display the characteristic features of pyridinium-based cations.^24^ The main profile consists of three contributions from the cation (Figure 7b): a high-energy peak at 288.8 eV, attributed to carbon atoms directly bonded to the positively charged nitrogen in the pyridinium ring; an intermediate peak at 286.2 eV, corresponding to the remaining aromatic ring carbons; and a low-binding-energy peak at 284.6 eV, assigned to aliphatic sp^3^-hybridized carbon atoms in the alkyl chain. The peak signals in these regions can also arise from adventitious carbon contamination on the substrate. In fact, the main peaks in the C 1s spectra exhibit similar intensities for both [C_2_py]­[NTf_2_] and [C_9_py]­[NTf_2_], despite the longer alkyl chain in [C_9_py], which would be expected to contribute a larger aliphatic signal. This observation suggests that contributions from adventitious carbon, either present on the substrate prior to deposition or introduced during XPS analysis, may partially obscure the expected differences in the cation-derived peaks. Additionally, the C 1s spectra exhibit a peak at 292.8 eV, corresponding to the carbon atoms in the −CF_3_ groups of the [NTf_2_]^−^ anion. The N 1s spectra (Figure 7c and i) show two well-defined peaks for all samples. The peak at higher binding energy, centered at 402.3 eV, is assigned to the nitrogen atom of the pyridinium cation, whereas the peak at lower binding energy, at 399.3 eV, originates from the nitrogen atom of the [NTf_2_]^−^ anion. The large energy separation between these two peaks reflects the more electropositive character of the cationic nitrogen compared to the anionic nitrogen, in agreement with previous XPS studies of pyridinium-based ILs.^24^ No additional nitrogen-related features are detected, confirming the absence of chemical degradation or ion dissociation. Small variations in the relative intensities between [C_2_py]­[NTf_2_] and [C_9_py]­[NTf_2_] can be attributed to differences in ion-pair orientation and near-surface composition rather than changes in chemical state. On ITO, the substrate-related O 1s peak (Figure 7d) appears at 529.9 eV for both ILs, while the oxygen contributions associated with the sulfonyl groups of the [NTf_2_]^−^ anion are observed at 532.2 eV. In contrast, on Au/ITO/glass substrates (Figure 7j), the O 1s spectra display exclusively the contribution from the anion, as expected due to the absence of oxygen in the Au surface. The F 1s spectra (Figures 7e and k) exhibit a single symmetric peak centered at 688.5 eV, characteristic of the CF_3_ groups of the [NTf_2_]^−^ anion, with no evidence of additional peaks or significant shifts. Similarly, the S 2p spectra (Figure 7f and l) show the expected spin–orbit doublet, with S 2p_3/2_ at 168.7 eV and S 2p_1/2_ at 169.8 eV, confirming the chemical integrity of the anion.

**Figure 7:**
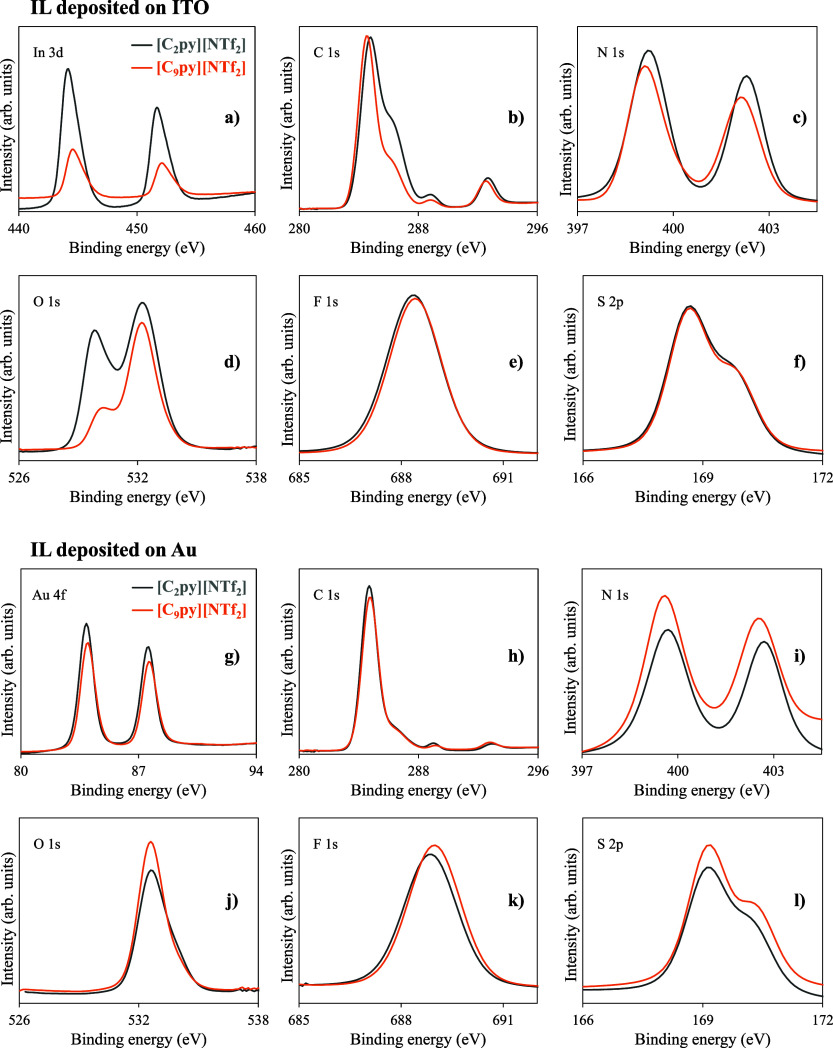
High-resolution XPS spectra of [C_2_py]­[NTf_2_] (brown) and [C_9_py]­[NTf_2_] (orange) thin films deposited on ITO/glass (a–f) and Au/ITO/glass (g–l) substrates. The spectra correspond to In 3d (a), C 1s (b, h), N 1s (c, i), O 1s (d, j), F 1s (e, k), and S 2p (f, l). Data correspond to films with a deposition amount of 14.6 ± 0.5 μg·cm^–2^.

Overall, the XPS analysis confirms that both [C_2_py]­[NTf_2_] and [C_9_py]­[NTf_2_] films retain their chemical integrity upon deposition, with the cation and anion components clearly resolved. The longer alkyl chain in [C_9_py]­[NTf_2_] leads to more effective substrate coverage, consistent with the enhanced lateral spreading and droplet coalescence observed in the SEM studies.

## Conclusions

This work represents the first systematic exploration of vacuum deposition across an extended series of pyridinium-based ionic liquids and provides crucial insights for the rational design of IL coatings and functional thin films. This study demonstrates that the morphology and wetting behavior of vacuum-deposited pyridinium-based IL films are strongly governed by the cation alkyl chain length and substrate type. As revealed by SEM studies, longer chains promote enhanced lateral spreading and more continuous surface coverage, while the odd–even alternation in chain length modulates droplet organization and coalescence dynamics. The preservation of chemical integrity, as confirmed by FTIR and XPS, underscores the robustness of these films under vacuum deposition. Overall, these insights provide a mechanistic framework for understanding and controlling the vapor deposition of thin ionic liquid films, offering guidance for their design in a wide range of technological applications, from organic electronics and sensors to lubrication and surface modification.

## Supplementary Material



## Notes

Details on the relevant properties of the ionic liquids studied, the vacuum deposition methodology and conditions, optical and SEM images of IL films on ITO and Au substrates, and XPS and FTIR characterization data are provided (PDF)
