# Supplementary material for: Dependency of Morphology and Wetting on Alkyl Chain Length in Vacuum-Evaporated [C n py][NTf2] (n = 2–9) Pyridinium Ionic Liquid Films
Source: Langmuir. 2026 Mar 17;42(12):8969–81. doi: 10.1021/acs.langmuir.6c00729 (PMC13045020; doi:10.1021/acs.langmuir.6c00729)
Supplement: Supplementary file 1 [file la6c00729_si_001.pdf]

Supporting Information (SI)

Dependency of Morphology and Wetting on Alkyl Chain Length in Vacuum-Evaporated [$C_n$py][NTf_2] (n = 2–9) Pyridinium Ionic Liquid Films

Soraia R. M. R. Silva,† João M. S. Pereira,† Oleksandr Bondarchuk,‡ Mauro C. C. Ribeiro,# Luís M. N. B. F. Santos,† and José C. S. Costa,†*

*Corresponding author

Email: jose.costa@fc.up.pt

† CIQUP/Institute of Molecular Sciences (IMS), Departamento de Química e Bioquímica, Faculdade de Ciências, Universidade do Porto, Rua do Campo Alegre s/n, 4169-007 Porto, Portugal.

‡ International Iberian Nanotechnology Laboratory, Av. Mestre José Veiga, s/n, 4715-330, Braga, Portugal; SPIN-Lab Centre for Microscopic Research on Matter, University of Silesia in Katowice, 75 Pułku Piechoty Str. 1A, Chorzów 41-500, Poland; Institute of Chemistry, University of Silesia in Katowice, 9 Szkolna Str., 40-006 Katowice, Poland.

# Laboratório de Espectroscopia Molecular, Instituto de Química, Universidade de São Paulo, 5513-970 São Paulo, SP, Brazil.

Details on the relevant properties of the ionic liquids studied, the vacuum deposition methodology and conditions, optical and SEM images of IL films on ITO and Au substrates, and XPS and FTIR characterization data are provided.

Index

1. Relevant Properties of the ILs Studied P. S5

2. Experimental Conditions for Film Deposition P. S6

3. Thin Film Morphology P. S7

4. Infrared Absorption Spectroscopy P. S12

5. X-Ray Photoelectron Spectroscopy P. S15

References P. S18

List of Tables

Table S1. CAS registry number, molar mass, density, melting temperature, glass transition temperature, viscosity, and surface tension values for the ionic liquids studied. P. S5

Table S2. Density- and viscosity-derived parameters for [C_3py][NTf_2], [C_4py][NTf_2], [C_5py][NTf_2], and [C_6py][NTf_2], including thermal expansion coefficient and Vogel temperature. P. S5

Table S3. Experimental conditions used for the deposition of [C_4py][NTf_2] at different flow rates and thicknesses on ITO-coated glass and Au/ITO-coated glass substrates. P. S6

Table S4. Experimental conditions used for the deposition of pyridinium-based ionic liquids with different alkyl chain lengths and thicknesses on ITO-coated glass and Au/ITO-coated glass substrates. P. S6

List of Figures

Figure S1. Thermal expansion coefficient and Vogel temperature as a function of the alkyl chain length for the [$C_n$py][NTf_2] ionic liquids. P. S7

Figure S2. Schematic illustration of the typical mechanisms of nucleation and growth of ionic liquid films obtained by vapor deposition. P. S7

Figure S3. Morphology of the ITO-coated glass and Au/ITO-coated glass substrates: SEM and AFM images. P. S8

Figure S4. Optical microscopy images of [C_4py][NTf_2] films deposited on ITO-coated glass substrates at different flow rates. P. S9

Figure S5. Optical microscopy images of [C_4py][NTf_2] films deposited on Au/ITO-coated glass substrates at different flow rates. P. S10

Figure S6. Optical microscopy images of [$C_n$py][NTf_2] films deposited on ITO-coated glass and Au/ITO-coated glass substrates. Shown are films of [C_2py][NTf_2], [C_4py][NTf_2], [C_5py][NTf_2], [C_6py][NTf_2], [C_7py][NTf_2], [C_8py][NTf_2], and [C_9py][NTf_2]. P. S11

Figure S7. Infrared absorption spectra of [C_2py][NTf_2] in the bulk phase and as a thin film deposited on Au/ITO-coated glass. P. S12

Figure S8. Infrared absorption spectra of [C_8py][NTf_2] in the bulk phase and as a thin film deposited on Au/ITO-coated glass. P. S12

Figure S9. Infrared absorption spectra of [C_9py][NTf_2] in the bulk phase and as a thin film deposited on Au/ITO-coated glass. P. S13

Figure S10. Infrared absorption spectra of bulk [C_2py][NTf_2], [C_8py][NTf_2], and [C_9py][NTf_2]. P. S13

Figure S11. Infrared absorption spectra of [C_2py][NTf_2], [C_8py][NTf_2], and [C_9py][NTf_2] films deposited on ITO-coated glass and Au/ITO-coated glass substrates. P. S14

Figure S12. Infrared absorption spectra of [C_2py][NTf_2], [C_8py][NTf_2], and [C_9py][NTf_2] films deposited on Au/ITO-coated glass, highlighting the shift of the band near 1200 cm^{-1} with increasing alkyl chain length. P. S14

Figure S13. XPS survey spectra of the ITO-coated glass surface (substrate exposed to air). P. S15

Figure S14. XPS survey spectra of the ITO-coated glass surface after the removal of adventitious carbon. P. S15

Figure S15. XPS survey spectrum of [C_2py][NTf_2] deposited on ITO-coated glass. P. S16

Figure S16. XPS survey spectrum of [C_2py][NTf_2] deposited on Au/ITO-coated glass. P. S16

Figure S17. XPS survey spectrum of [C_9py][NTf_2] deposited on ITO-coated glass. P. S17

Figure S18. XPS survey spectrum of [C_9py][NTf_2] deposited on Au/ITO-coated glass. P. S17

1. Relevant Properties of the ILs Studied

Table S1. CAS registry number (CAS), molar mass (M), density (ρ), melting temperature (T_m), glass transition temperature (T_g), viscosity (η), and surface tension (γ) values for the ionic liquids studied.

Ionic Liquid	CAS	M / g·mol^{-1}	ρ (298K) / g·cm^{-3}	T_m / K	T_g / K	η (298K) / mPa·s	γ (298K) / mN·m^{-1}
[C_2py][NTf_2]	712354-97-7	388.3	1.5375[1]	303.65[1]	235.24[1]	39.4[2]	37.4[1]
[C_4py][NTf_2]	187863-42-9	416.4	1.4547[1]	299.05[1]	N/A	58.3[2]	33.4[1]
[C_5py][NTf_2]	N/A	430.4	1.4214[1]	272.84[1]	196.22[1]	71.9[2]	32.5[1]
[C_6py][NTf_2]	460983-97-5	444.4	1.3877[3]	276.4[3]	196.6[3]	84.5[3]	31.7[3]
[C_7py][NTf_2]	N/A	458.5	1.3541[4]	272[4]	197[4]	99.5[4]	N/A
[C_8py][NTf_2]	384347-06-2	472.5	1.3268[5]	261.15[6]	195.2[6]	114.3[5]	N/A
[C_9py][NTf_2]	N/A	486.5	N/A	N/A	N/A	N/A	N/A

N/A = not available

Table S2. Density- and viscosity-derived parameters for [C_3py][NTf_2], [C_4py][NTf_2], [C_5py][NTf_2], and [C_6py][NTf_2], including thermal expansion coefficient (α) and Vogel temperature (T_0).

Ionic Liquid	$10^4 \cdot \alpha$ (298 K)/ K^{-1}	T_o / K
[C_3py][NTf_2]	6.50 ± 0.01[7]	172.3 ± 0.2[7]
[C_4py][NTf_2]	6.44 ± 0.04[7]	168.5 ± 0.2[7]
[C_5py][NTf_2]	6.48 ± 0.04[7]	168.0 ± 0.3[7]
[C_6py][NTf_2]	6.41 ± 0.04[7]	164.4 ± 0.7[7]

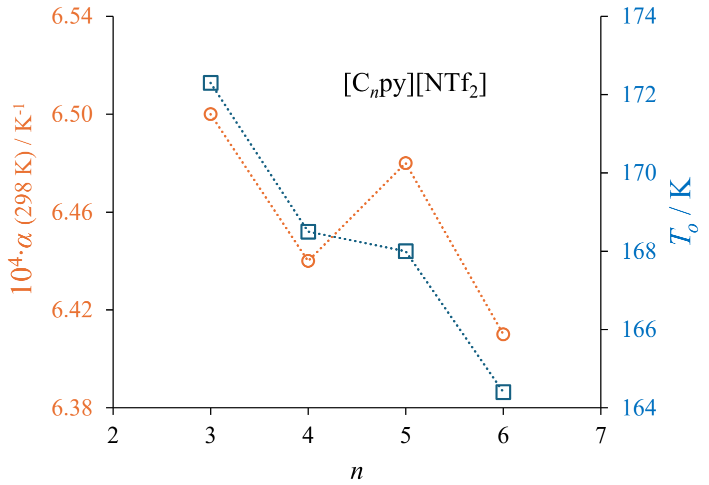

Figure S1. Thermal expansion coefficient (α) and Vogel temperature (T_0) as a function of the alkyl chain length (n) for the [$C_n$py][NTf_2] ionic liquids.

2. Experimental Conditions for Film Deposition

Table S3. Experimental conditions used for the deposition of [C_4py][NTf_2] at different flow rates and thicknesses on ITO-coated glass and Au/ITO-coated glass substrates.

Ionic Liquid	M / g·mol^{-1}	ρ (298K) / g·cm^{-3}	$T_{effusion}$ / K	φ / Å·s^{-1}	$t_{deposition}$ / s	m_{film} / µg·cm^{-2}	l_{film} / nm
[C_4py][NTf_2]	416.4	1.4547	483.15 ± 0.05	0.08 ± 0.01	6665	7.3 ± 0.5	50 ± 5
			503.15 ± 0.05	0.25 ± 0.01	1971		
			523.15 ± 0.05	0.54 ± 0.01	923		
			533.15 ± 0.05	1.10 ± 0.01	455		
			483.15 ± 0.05	0.09 ± 0.01	10752	14.6 ± 0.5	100 ± 10
			503.15 ± 0.05	0.27 ± 0.01	3712		
			523.15 ± 0.05	0.76 ± 0.01	1314		
			533.15 ± 0.05	1.18 ± 0.01	845		

Table S4. Experimental conditions used for the deposition of pyridinium-based ionic liquids with different alkyl chain lengths and thicknesses on ITO-coated glass and Au/ITO-coated glass substrates.

Ionic Liquid	M / g·mol^{-1}	ρ (298K) / g·cm^{-3}	$T_{effusion}$ / K	φ / Å·s^{-1}	$t_{deposition}$ / s	m_{film} / µg·cm^{-2}	l_{film} / nm
[C_2py][NTf_2]	388.3	1.5375	503.15 ± 0.05	0.19 ± 0.01	2517	7.3 ± 0.5	50 ± 5
					4997	14.6 ± 0.5	100 ± 10
[C_4py][NTf_2]	416.4	1.4547	503.15 ± 0.05	0.25 ± 0.01	1971	7.3 ± 0.5	50 ± 5
					3712	14.6 ± 0.5	100 ± 10
[C_5py][NTf_2]	430.4	1.4214	508.15 ± 0.05	0.27 ± 0.01	1874	7.3 ± 0.5	50 ± 5
					3895	14.6 ± 0.5	100 ± 10
[C_6py][NTf_2]	444.4	1.3877	508.15 ± 0.05	0.40 ± 0.01	1320	7.3 ± 0.5	50 ± 5
					2630	14.6 ± 0.5	100 ± 10
[C_7py][NTf_2]	458.5	1.3541	508.15 ± 0.05	0.31 ± 0.01	2032	7.3 ± 0.5	50 ± 5
					3425	14.6 ± 0.5	100 ± 10
[C_8py][NTf_2]	472.5	1.3268	508.15 ± 0.05	0.41 ± 0.01	1350	7.3 ± 0.5	50 ± 5
					2705	14.6 ± 0.5	100 ± 10
[C_9py][NTf_2]	486.5	1.3040	508.15 ± 0.05	0.42 ± 0.01	1335	7.3 ± 0.5	50 ± 5
					2680	14.6 ± 0.5	100 ± 10

3. Thin Film Morphology

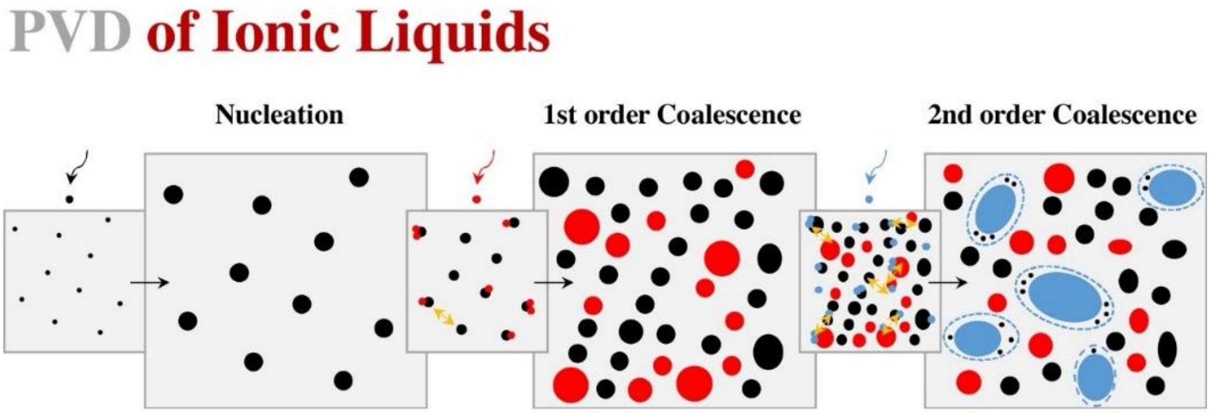

Figure S2. Schematic illustration of the typical mechanisms of nucleation and growth of ionic liquid films obtained by vapor deposition: minimum free area to promote nucleation (MFAN); first-order coalescence; second-order coalescence.[8]

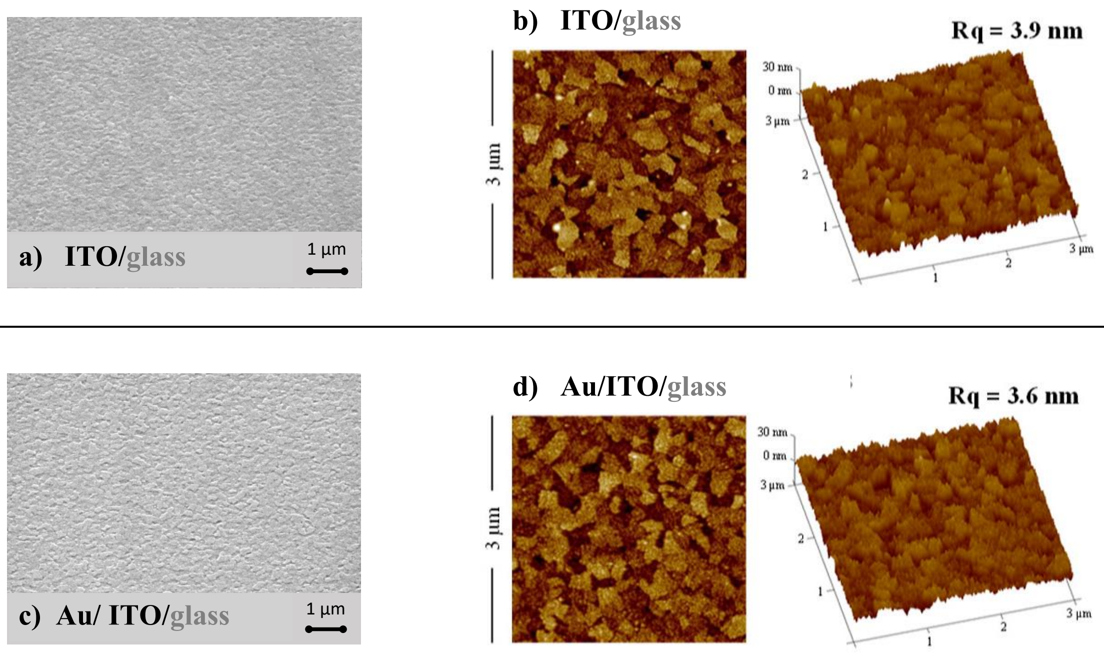

Figure S3. Morphology of the substrates: ITO-coated glass (a and b) and Au/ITO-coated glass (c and d). SEM images (a and c) were acquired using high-resolution scanning electron microscopy (SEM) with a secondary electron detector, and AFM images (b and d) were recorded in tapping mode. The ITO/glass substrates, measuring 10 mm × 10 mm × 1.1 mm, were commercially obtained from Praezisions Glas & Optik GmbH. The ITO films have an approximate thickness of 180 nm. To prepare the metal surfaces (Au/ITO), the ITO substrates underwent a sputtering process, depositing a gold film with a thickness of 100 nm onto the ITO surfaces.

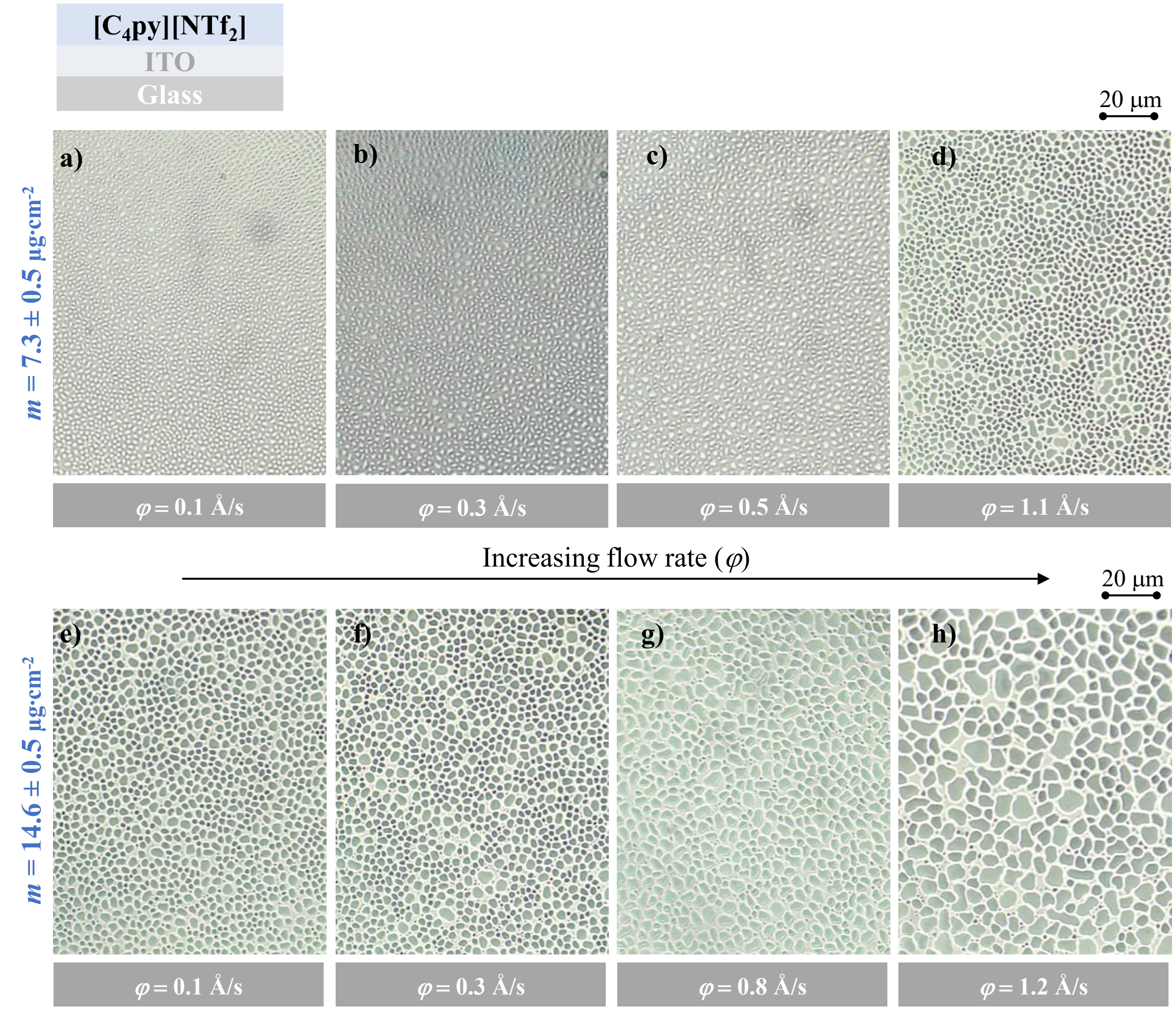

Figure S4. Optical microscopy images (a-h) of $[C_4py][NTf_2]$ films deposited on ITO-coated glass substrates at different flow rates (φ). Experimental data are shown for deposition amounts of 7.3 ± 0.5 μg·cm^{-2} (thickness of ≈ 50 nm, micrographs a, b, c, and d) and 14.6 ± 0.5 μg·cm^{-2} (thickness of ≈ 100 nm, micrographs e, f, g, and h and histograms m, n, o, and p). Samples were visualized immediately after deposition.

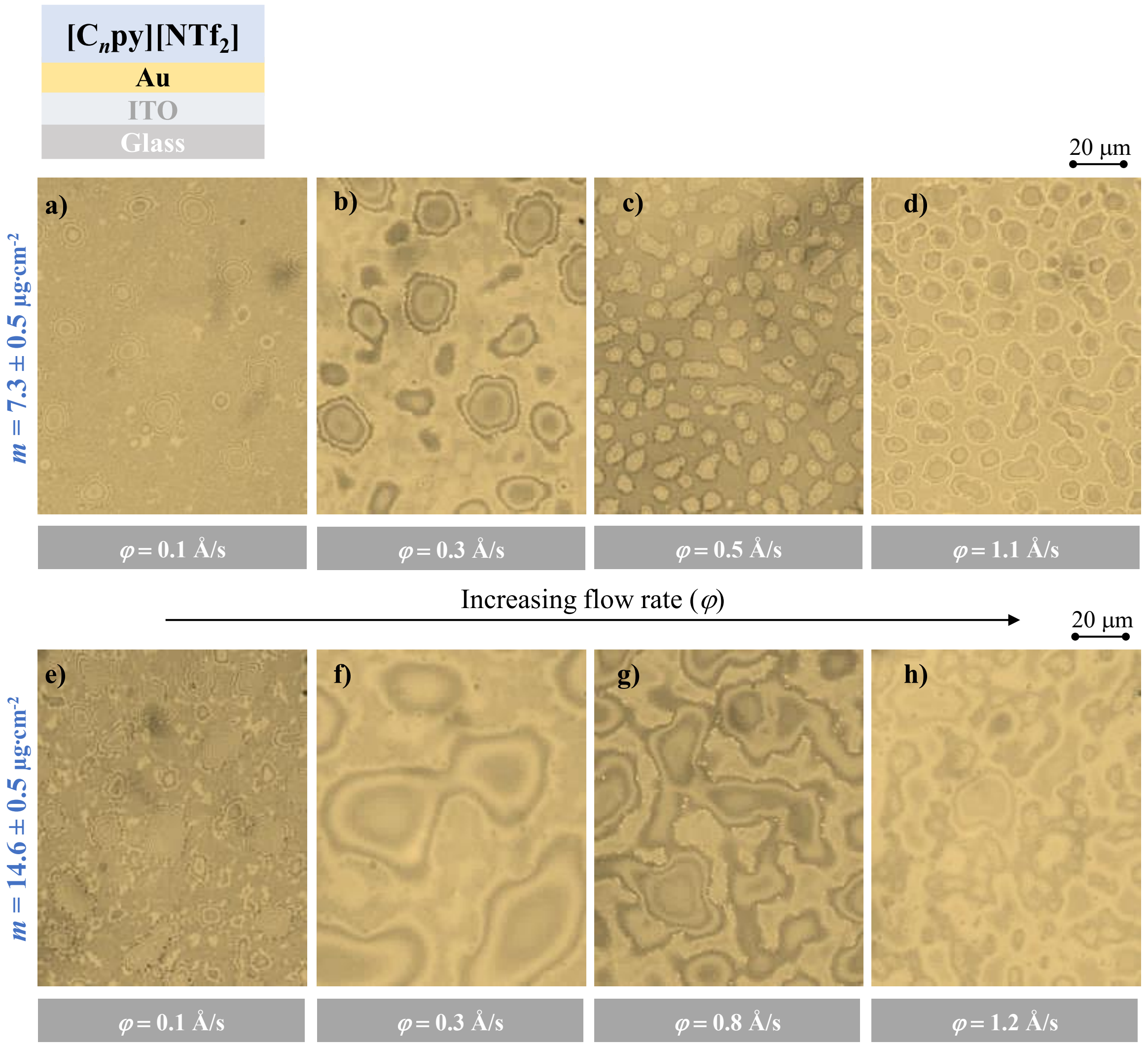

Figure S5. Optical microscopy images (a-h) of [C_4py][NTf_2] films deposited on Au/ITO-coated glass substrates at different flow rates (φ). Experimental data are shown for deposition amounts of 7.3 ± 0.5 $\mu g \cdot cm^{-2}$ (thickness of ≈ 50 nm, micrographs a, b, c, and d) and 14.6 ± 0.5 $\mu g \cdot cm^{-2}$ (thickness of ≈ 100 nm, micrographs e, f, g, and h and histograms m, n, o, and p). Samples were visualized immediately after deposition.

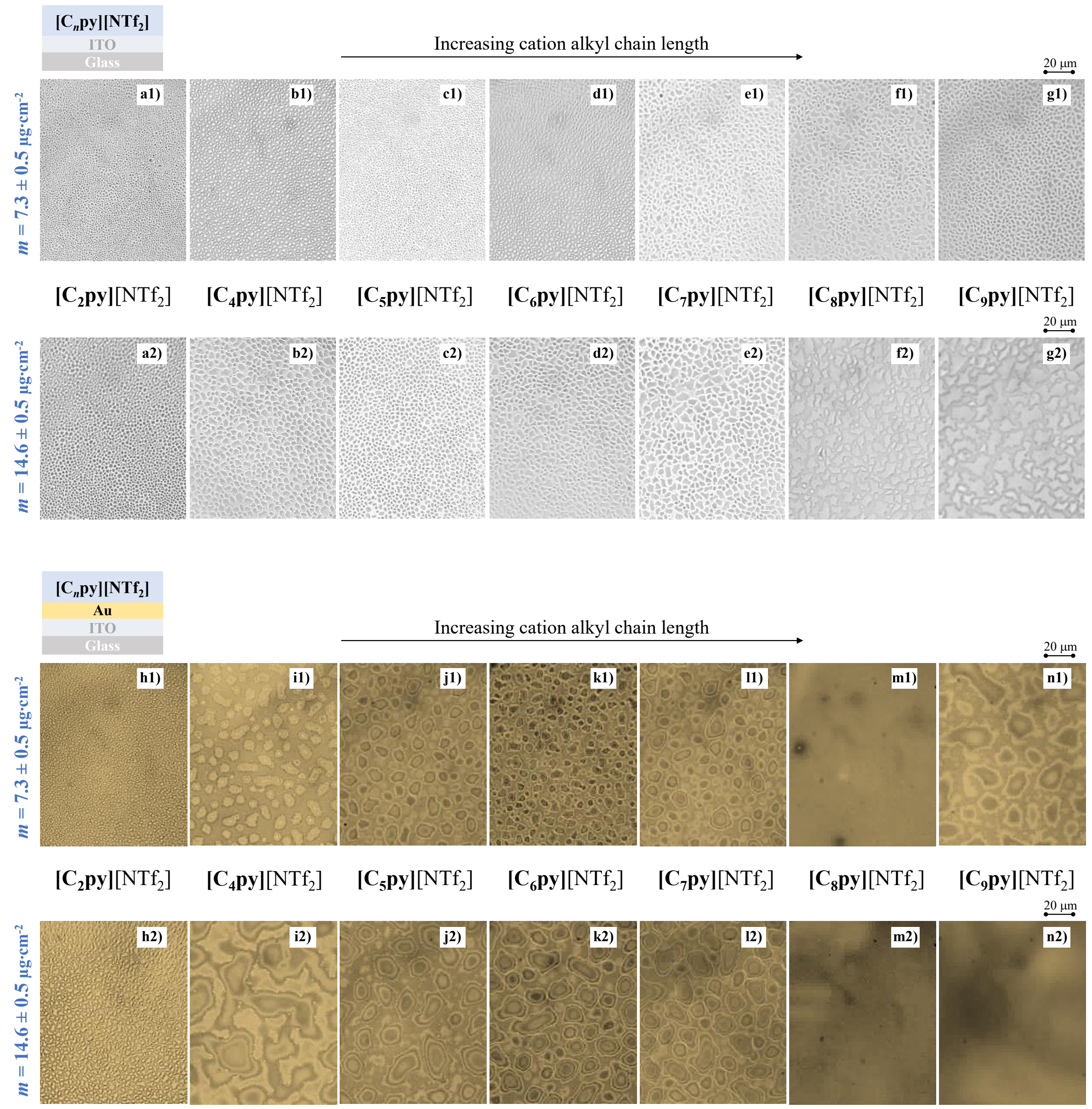

Figure S6. Optical microscopy images of [$C_n$py][NTf_2] films deposited on ITO-coated glass (a–g) and Au/ITO-coated glass (h–n) substrates. Shown are films of [C_2py][NTf_2] (a1, a2, h1, h2), [C_4py][NTf_2] (b1, b2, i1, i2), [C_5py][NTf_2] (c1, c2, j1, j2), [C_6py][NTf_2] (d1, d2, k1, k2), [C_7py][NTf_2] (e1, e2, l1, l2), [C_8py][NTf_2] (f1, f2, m1, m2), and [C_9py][NTf_2] (g1, g2, n1, n2). Experimental data correspond to deposition amounts of 7.3 ± 0.5 µg·cm^{-2} (micrographs a1-g1 and h1-n1) and 14.6 ± 0.5 µg·cm^{-2} (micrographs a2-g2 and h2-n2), obtained at a constant deposition rate of φ = 0.3 Å·s^{-1}. Samples were visualized immediately after deposition.

4. Infrared Absorption Spectroscopy

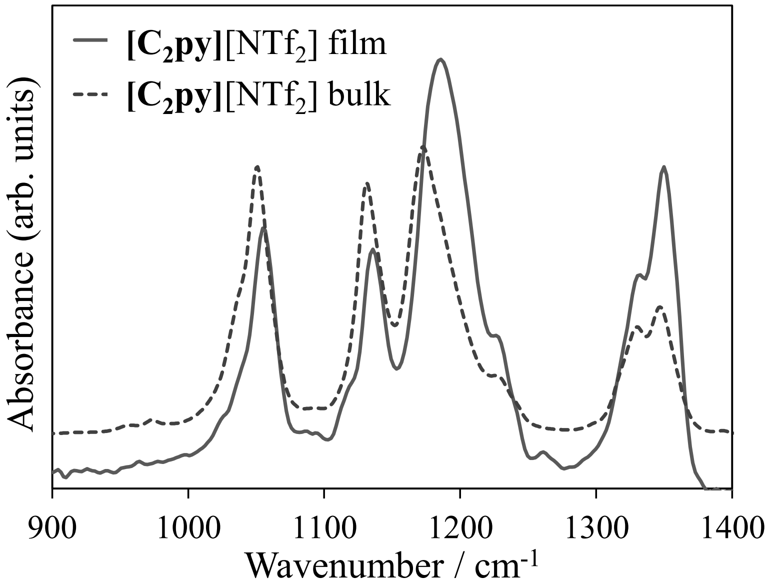

Figure S7. Infrared absorption spectra of $[C_2py][NTf_2]$ in the bulk phase and as a thin film deposited on Au/ITO-coated glass. The bulk spectrum was recorded in ATR mode by placing a drop of the ionic liquid directly on the ATR crystal. The thin film sample corresponds to a deposition amount of 14.6 ± 0.5 μg·cm^{-2}, and the spectrum was obtained by specular reflectance using a spectrometer operating in reflectance-absorption mode.

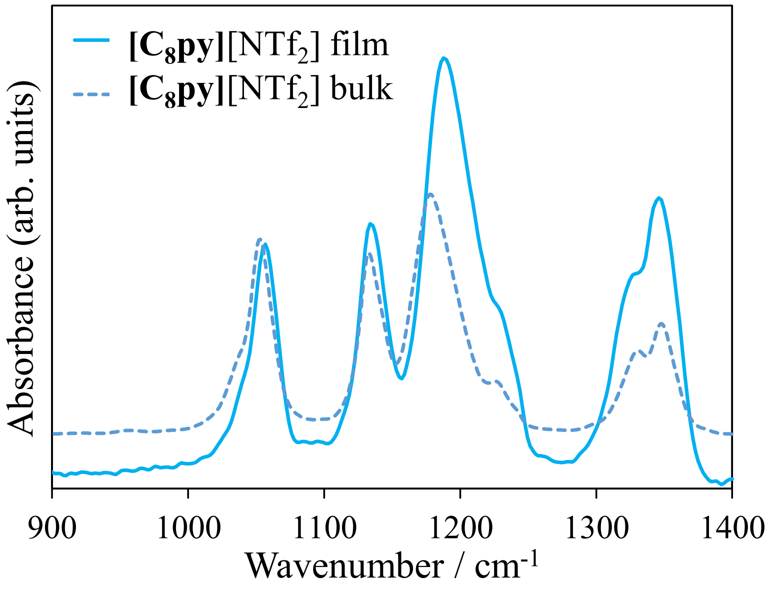

Figure S8. Infrared absorption spectra of $[C_8py][NTf_2]$ in the bulk phase and as a thin film deposited on Au/ITO-coated glass. The bulk spectrum was recorded in ATR mode by placing a drop of the ionic liquid directly on the ATR crystal. The thin film sample corresponds to a deposition amount of 14.6 ± 0.5 μg·cm^{-2}, and the spectrum was obtained by specular reflectance using a spectrometer operating in reflectance-absorption mode.

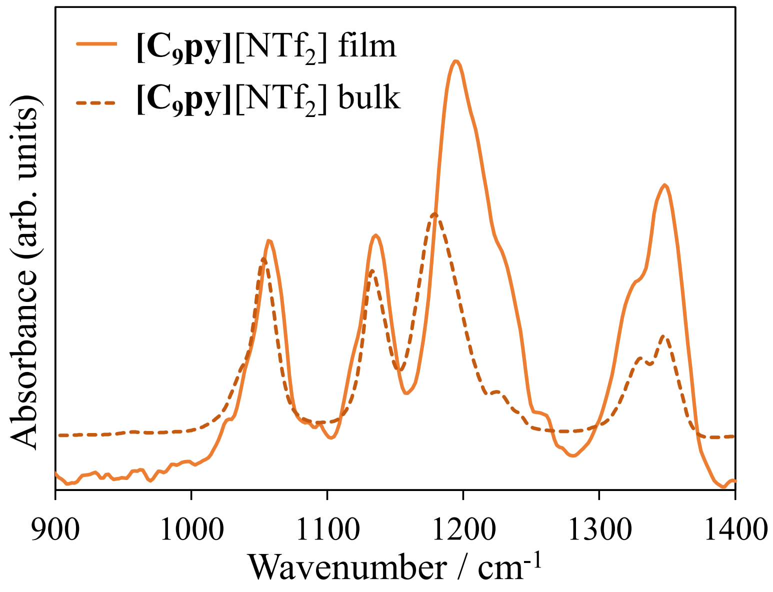

Figure S9. Infrared absorption spectra of [C_9py][NTf_2] in the bulk phase and as a thin film deposited on Au/ITO-coated glass. The bulk spectrum was recorded in ATR mode by placing a drop of the ionic liquid directly on the ATR crystal. The thin film sample corresponds to a deposition amount of 14.6 ± 0.5 μg·cm^{-2}, and the spectrum was obtained by specular reflectance using a spectrometer operating in reflectance-absorption mode.

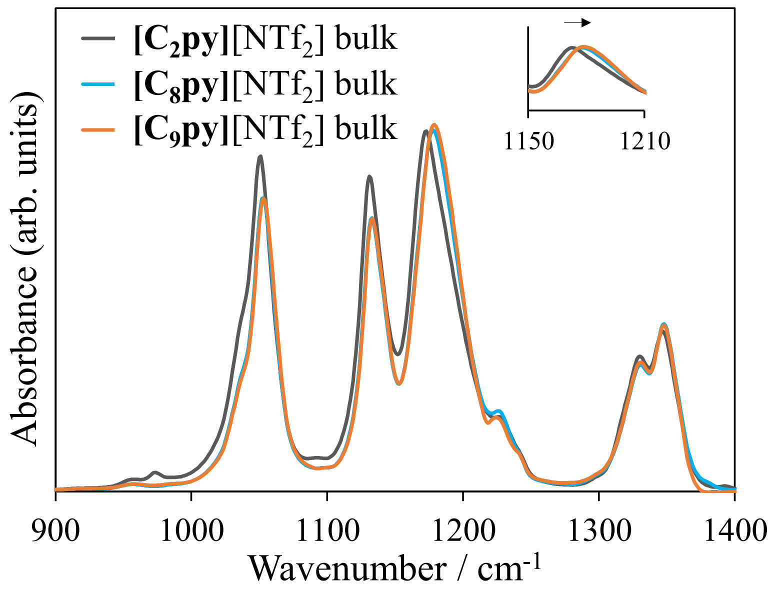

Figure S10. Infrared absorption spectra of bulk [C_2py][NTf_2], [C_8py][NTf_2], and [C_9py][NTf_2]. Bulk spectra were recorded in ATR mode by placing a drop of the ionic liquid directly on the ATR crystal.

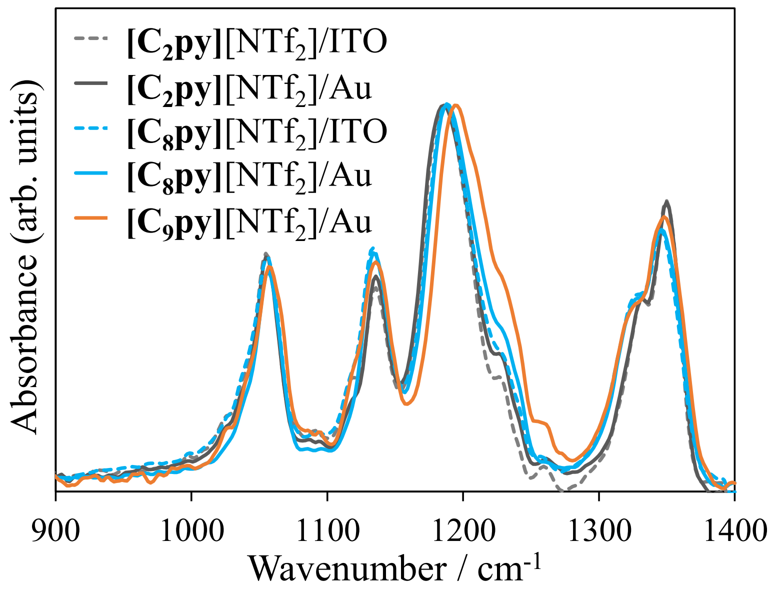

Figure S11. Infrared absorption spectra of [C_2py][NTf_2], [C_8py][NTf_2], and [C_9py][NTf_2] films deposited on ITO-coated glass and Au/ITO-coated glass substrates. The thin film samples correspond to a deposition amount of 14.6 ± 0.5 μg·cm^{-2}, and the spectra were obtained by specular reflectance using a spectrometer operating in reflectance-absorption mode.

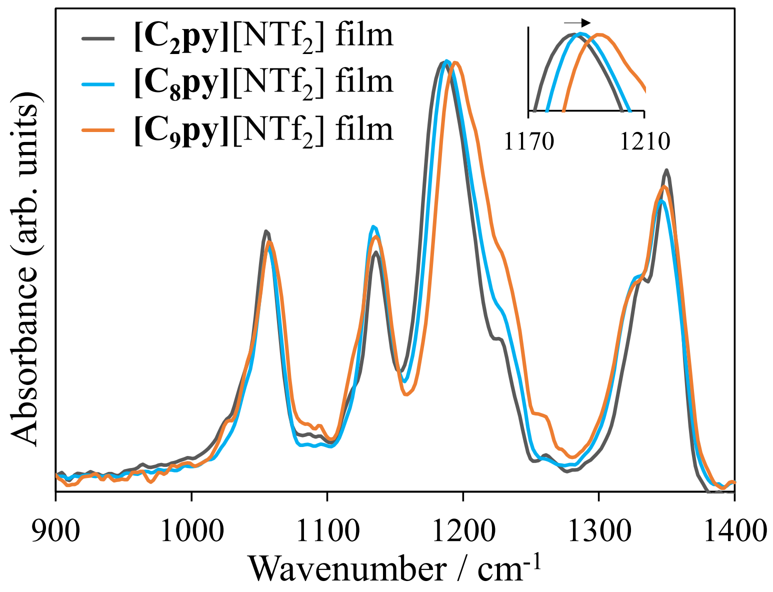

Figure S12. Infrared absorption spectra of [C_2py][NTf_2], [C_8py][NTf_2], and [C_9py][NTf_2] films deposited on Au/ITO-coated glass, highlighting the shift of the band near 1200 cm^{-1} with increasing alkyl chain length. The thin film samples correspond to a deposition amount of 14.6 ± 0.5 μg·cm^{-2}, and the spectra were obtained by specular reflectance using a spectrometer operating in reflectance-absorption mode.

5. X-Ray Photoelectron Spectroscopy (XPS)

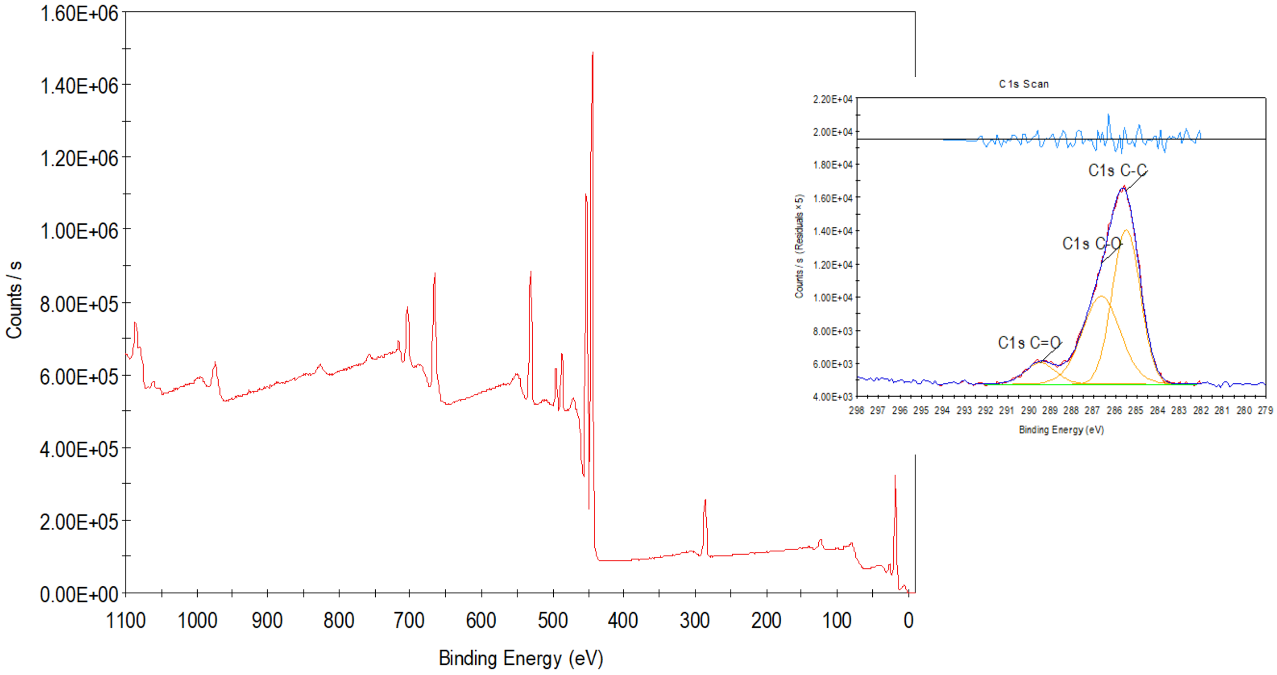

Figure S13. XPS survey spectra of the ITO-coated glass (substrate exposed to air). The C1s spectrum reveals adventitious carbon contamination on the surface, with detected C–C, C–O, and C=O components.

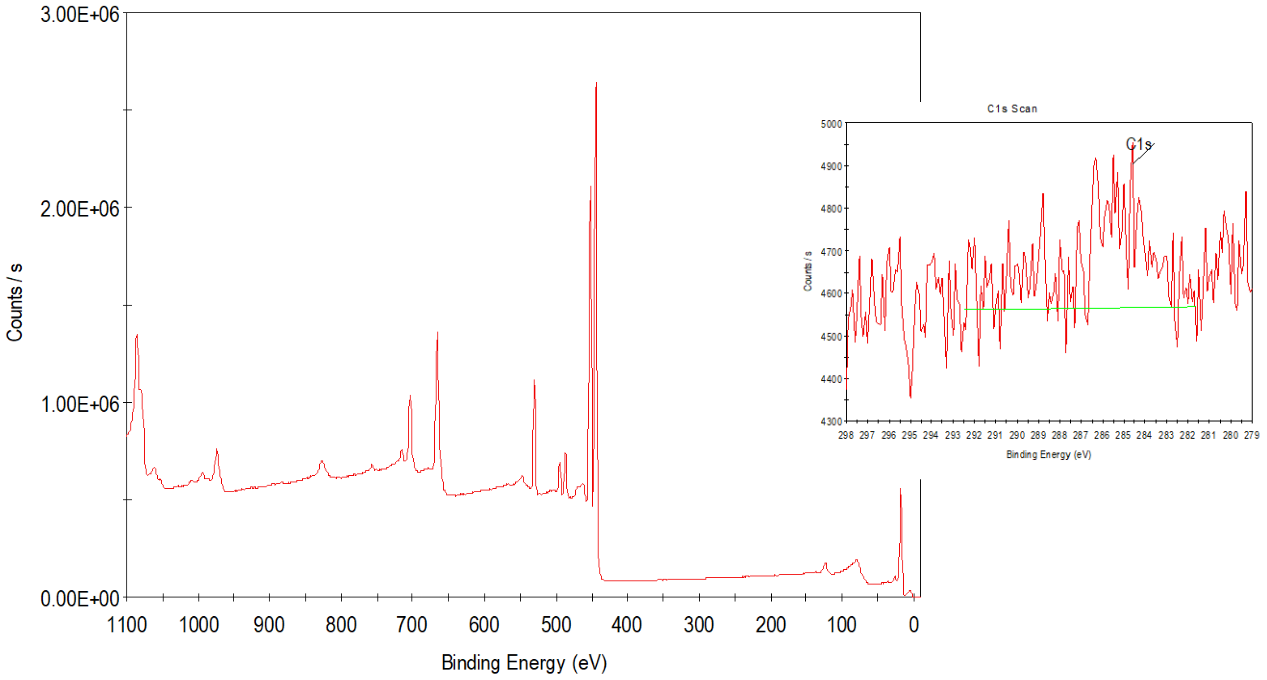

Figure S14. XPS survey spectra of the ITO-coated glass after the removal of adventitious carbon by argon sputtering.

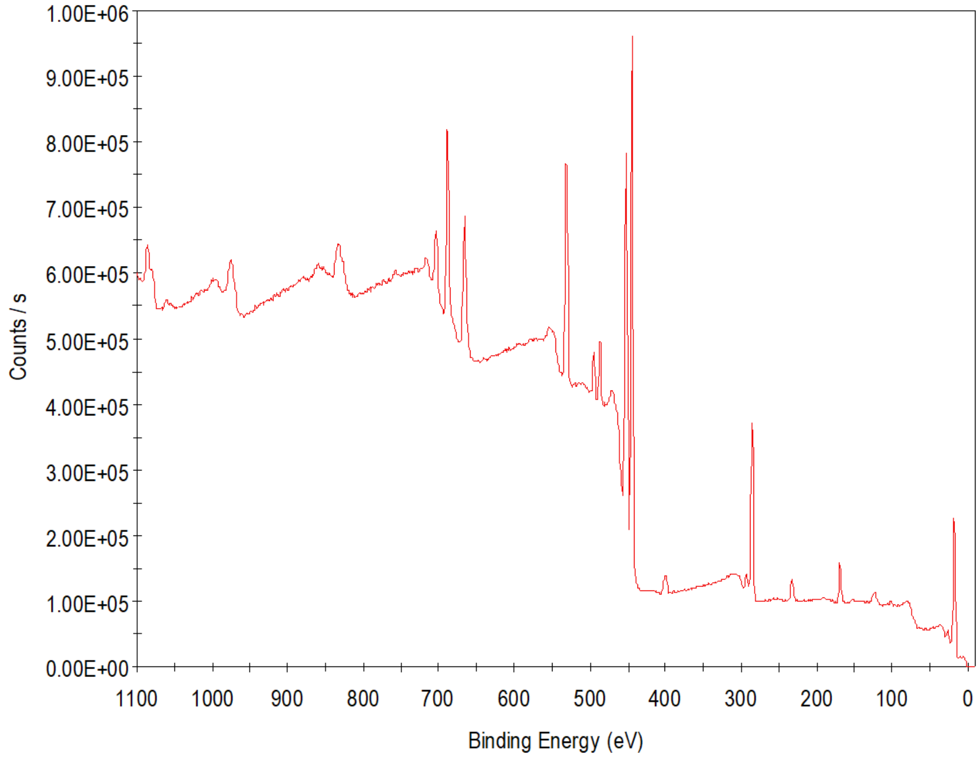

Figure S15. XPS survey spectrum of [C_2py][NTf_2] deposited on ITO-coated glass.

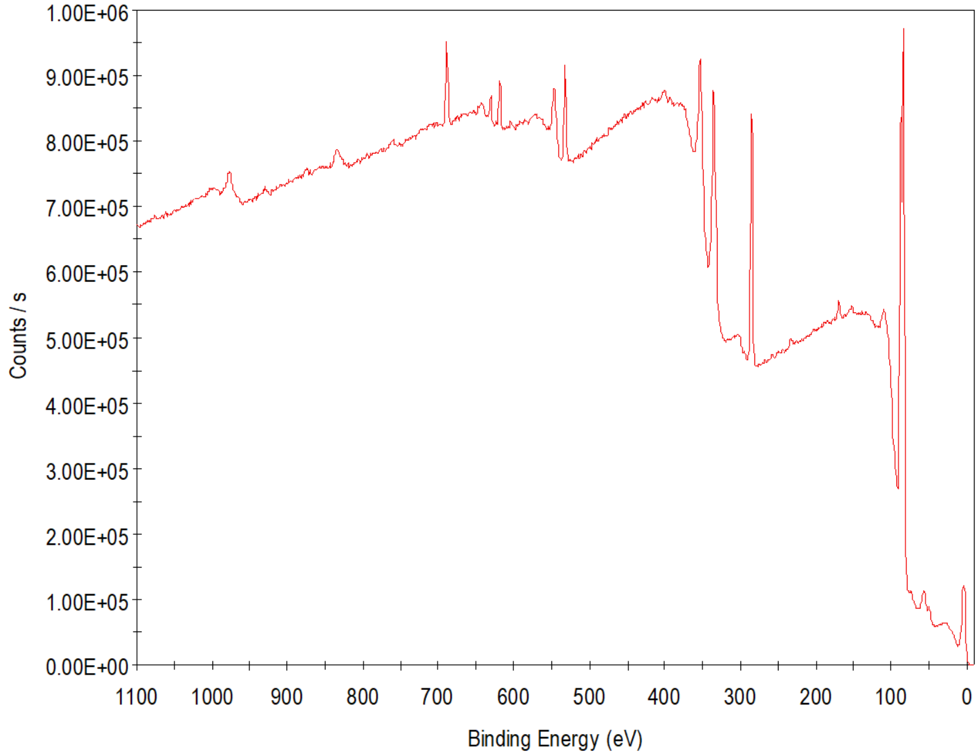

Figure S16. XPS survey spectrum of [C_2py][NTf_2] deposited on Au/ITO-coated glass.

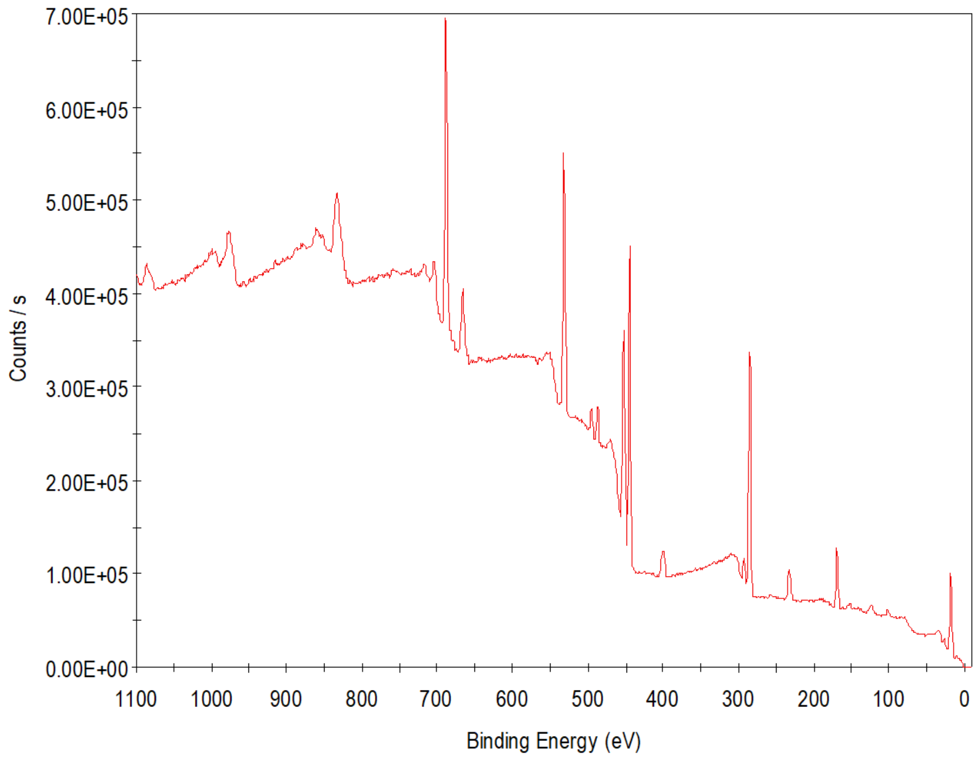

Figure S17. XPS survey spectrum of [C_9py][NTf_2] deposited on ITO-coated glass.

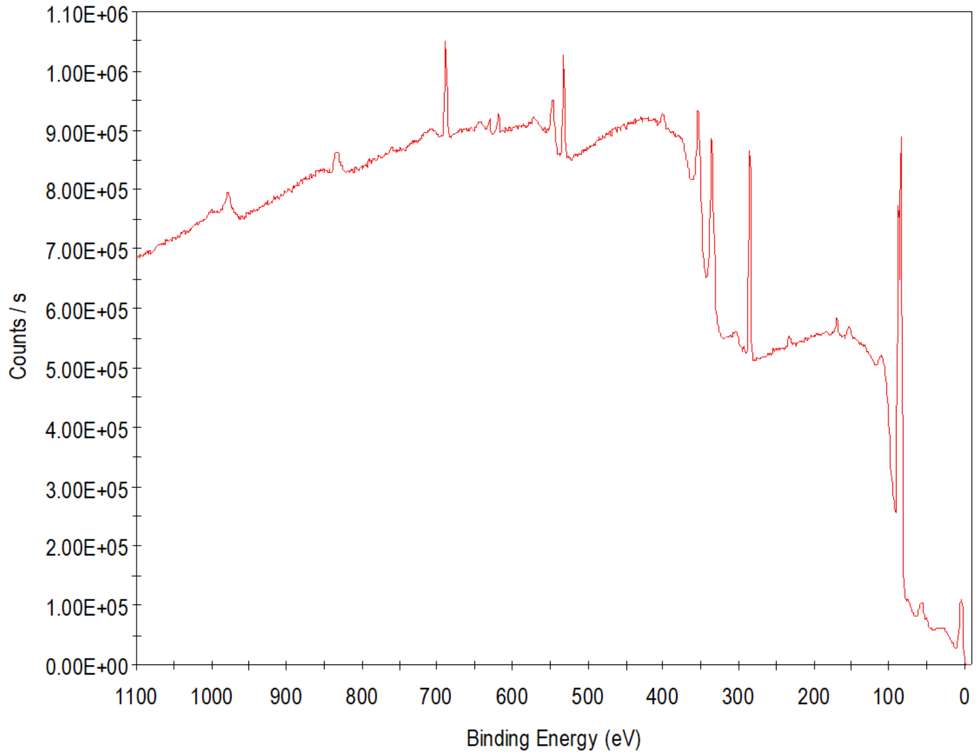

Figure S18. XPS survey spectrum of [C_9py][NTf_2] deposited on Au/ITO-coated glass.

References

(1) Liu, Q.-S.; Yang, M.; Yan, P.-F.; Liu, X.-M.; Tan, Z.-C.; Welz-Biermann, U. Density and Surface Tension of Ionic Liquids [$C_n$py][NTf_2] (n = 2, 4, 5). *J. Chem. Eng. Data* **2010**, *55*, 4928–4930.

(2) Liu, Q.-S.; Yan, P.-F.; Miao, Y.; Tan, Z.-C.; Li, C.-P.; Welz-Biermann, U. Dynamic Viscosity and Conductivity of Ionic Liquids [$C_n$py][NTf_2] (n = 2, 4, 5). *Acta Phys.-Chim. Sin.* **2011**, *27*, 2762–2766.

(3) Liu, Q.-S.; Yang, M.; Li, P.-P.; Sun, S.-S.; Welz-Biermann, U.; Tan, Z.-C.; Zhang, Q.-G. Physicochemical Properties of Ionic Liquids [C_3py][NTf_2] and [C_6py][NTf_2]. *J. Chem. Eng. Data* **2011**, *56*, 4094–4101.

(4) Zeng, S.; Wang, J.; Bai, L.; Wang, B.; Gao, H.; Shang, D.; Zhang, X.; Zhang, S. Highly Selective Capture of CO_2 by Ether-Functionalized Pyridinium Ionic Liquids with Low Viscosity. *Energy Fuels* **2015**, *29*, 6039–6048.

(5) Yunus, N. M.; Mutalib, M. I. A.; Man, Z.; Bustam, M. A.; Murugesan, T. Thermophysical Properties of 1-Alkylpyridinum Bis(trifluoromethylsulfonyl)imide Ionic Liquids. *J. Chem. Thermodyn.* **2010**, *42*, 491–495.

(6) Papaiconomou, N.; Salminen, J.; Lee, J.-M.; Prausnitz, J. M. Physicochemical Properties of Hydrophobic Ionic Liquids Containing 1-Octylpyridinium, 1-Octyl-2-methylpyridinium, or 1-Octyl-4-methylpyridinium Cations. *J. Chem. Eng. Data* **2007**, *52*, 833–840.

(7) Miranda, C. F. P. Effect of Structure on the Thermodynamic and Transport Properties of Ionic Liquids, **2025**; PhD Thesis; Repositório Aberto da Universidade do Porto. https://repositorio-aberto.up.pt/handle/10216/166857 (accessed Mar 2, 2026).

(8) Costa, J. C. S.; Coelho, A. F. S. M. G.; Mendes, A.; Santos, L. M. N. B. F. Nucleation and Growth of Microdroplets of Ionic Liquids Deposited by Physical Vapor Method onto Different Surfaces. *Appl. Surf. Sci.* **2018**, *428*, 242–249.
